# Genotoxic stress-activated DNA-PK-p53 cascade and autophagy cooperatively induce ciliogenesis to maintain the DNA damage response

**DOI:** 10.1038/s41418-020-00713-8

**Published:** 2021-01-18

**Authors:** Ting-Yu Chen, Bu-Miin Huang, Tang K. Tang, Yu-Ying Chao, Xiao-Yi Xiao, Pei-Rong Lee, Li-Yun Yang, Chia-Yih Wang

**Affiliations:** 1 Department of Cell Biology and Anatomy, College of Medicine, National Cheng Kung University, Tainan, Taiwan, ROC; 2 Institute of Basic Medical Sciences, College of Medicine, National Cheng Kung University, Tainan, Taiwan, ROC; 3 Department of Medical Research, China Medical University Hospital, China Medical University, Taichung, Taiwan, ROC; 4 Institute of Biomedical Sciences, Academia Sinica, Taipei, Taiwan, ROC

**Keywords:** DNA-PK, p53, Primary cilium, Autophagy, Genotoxic stress, DNA damage response, Cell biology, Molecular biology

## Abstract

The DNA-PK maintains cell survival when DNA damage occurs. In addition, aberrant activation of the DNA-PK induces centrosome amplification, suggesting additional roles for this kinase. Here, we showed that the DNA-PK-p53 cascade induced primary cilia formation (ciliogenesis), thus maintaining the DNA damage response under genotoxic stress. Treatment with genotoxic drugs (etoposide, neocarzinostatin, hydroxyurea, or cisplatin) led to ciliogenesis in human retina (RPE1), trophoblast (HTR8), lung (A459), and mouse Leydig progenitor (TM3) cell lines. Upon genotoxic stress, several DNA damage signaling were activated, but only the DNA-PK-p53 cascade contributed to ciliogenesis, as pharmacological inhibition or genetic depletion of this pathway decreased genotoxic stress-induced ciliogenesis. Interestingly, in addition to localizing to the nucleus, activated DNA-PK localized to the base of the primary cilium (mother centriole) and daughter centriole. Genotoxic stress also induced autophagy. Inhibition of autophagy initiation or lysosomal degradation or depletion of ATG7 decreased genotoxic stress-induced ciliogenesis. Besides, inhibition of ciliogenesis by depletion of IFT88 or CEP164 attenuated the genotoxic stress-induced DNA damage response. Thus, our study uncovered the interplay among genotoxic stress, the primary cilium, and the DNA damage response.

## Introduction

Cells are continuously exposed to several stresses from endogenous and exogenous sources. To maintain physiological homeostasis, a diverse range of signaling pathways are activated in cells. In vertebrates, members of the phosphatidylinositol 3-kinase-related kinase (PI3KK) superfamily, including ataxia telangiectasia, mutated (ATM), ataxia telangiectasia, mutated and Rad3-related (ATR) and DNA-dependent protein kinase (DNA-PK), initiate signaling in response to DNA damage [1].

DNA-PK is a heterotrimeric complex composed of a catalytic subunit, DNA-PKcs, and two regulatory subunits, Ku70 and Ku80 [2]. Once DNA double-strand breaks occur, Ku heterodimers, the DNA-binding component of DNA-PK, bind to the broken ends of DNA and recruit DNA-PKcs to form an active complex for DNA repair [3, 4]. Thus, DNA-PK plays an important role in the maintenance of genomic stability. DNA-PK-mediated p53 activation maintains cell survival [5]. In response to ionizing radiation, DNA-PK participates in p53 accumulation [6]. Dietary restriction activates DNA-PK-p53 cascade thus suppressing tumor grow [7]. Thus, the DNA-PK-p53 cascade apparently aims to arrest cell cycle progression and prevent tumorigenesis.

The centrosome is composed of two centrioles, the mother and daughter centrioles, and surrounding pericentriolar material [8] and is the main microtubule organizing center for orchestrating microtubule arrays and the mitotic apparatus. It also serves as the base for primary cilium growth [9]. The primary cilium is an immotile, microtubule-based protrusion from the mother centriole that mainly functions as a cellular antenna to sense environmental signaling [10]. This protrusion is composed of a central microtubule-based axoneme and the surrounding ciliary membrane. The microtubules of the axoneme are highly acetylated, which stabilizes the axoneme, and several signaling receptors localize to the ciliary membrane to transduce environmental cues [11]. Thus, the precise control of ciliogenesis is important for maintaining normal growth and differentiation.

Autophagy is a lysosomal degradation process whereby cells degrade and reutilize old organelles and proteins to maintain metabolic homeostasis [12]. It also participates in ciliogenesis [13]. Autophagic flux requires the formation of a double-membraned vesicle, the autophagosome. Once this vesicles forms, it fuses with the lysosome to form an autolysosome, and lysosomal hydrolases degrade the contents within this acidic compartment. Unc-51-like kinase (ULK) 1 and 2 are required for the initiation of autophagy [14]; they activate the class III PI3 kinase (PI3K) complex to promote phagophore formation. ATG7-mediated signaling then promotes autophagosome formation [15]. ATG7 activates ATG12 to promote the formation of the autophagosome precursor and initiates the conversion of LC3 I to LC3 II, a tightly membrane-bound form of LC3, ultimately promoting autophagosome formation [15]. Once the autophagosome forms, it fuses with lysosomes to degrade cytoplasmic organelles.

The primary cilium plays important roles in development and differentiation. Here, we showed that the DNA-PK-p53 cascade induced primary cilia formation, thus maintaining the DNA damage response under genotoxic stress. Treatment with genotoxic drugs led to ciliogenesis in several cell lines. Upon genotoxic stress, the DNA-PK-p53 cascade contributed to ciliogenesis. Interestingly, genotoxic stress also induced autophagy for ciliogenesis. Thus, our study uncovered the interplay among genotoxic stress, the primary cilium, and the DNA damage response.

## Materials and methods

### Cell culture

Human immortalized retina pigmented epithelial (RPE1) and mouse Leydig progenitor (TM3) cell lines were grown in Dulbecco’s modified Eagle medium (DMEM)-F12, human immortalized trophoblast (HTR8) cells were grown in Roswell Park Memorial Institute (RPMI)-1640 medium, and human adenocarcinomic human alveolar basal epithelial (A459) cells were grown in DMEM. All culture media were supplemented with 10% fetal bovine serum, and all cell lines were cultured at 37 °C in a humidified atmosphere of 5% CO_2_. These cells were regularly examined for mycoplasma contamination by immunoblotting, immunofluorescence, and DAPI staining according to the guidelines.

### Drug treatments

Dorsomorphin (AMPKi, S7306, 5 μM), SBI-0206965 (ULK1i, SML 1540, 10 μM), Caffeine (C0750, 2 mM), Ku55933 (ATM inhibitor, SML1109, 10 μM), 3-methyladenine (3-MA, 5142-23-4, 5 mM), cytochalasin D (C2618, 5 μg/ml), NCS (N9162, 0.5 mg/ml), HU (H8627, 2 mM), 7-hydroxy staurosporine (UCN-01, U6508, 100 nM), CPT (232120, 5 μM), pifithrin-α (p53 inhibitor, 506170, 10 μM), and chloroquine (CQ, 50-63-5, 50 μM) were purchased from Sigma, St. Louis, MO. Bafilomycin-A1 (Baf.A1, BML-CM110, 10 nM) was purchased from Enzo, NY, USA. Chk2 inhibitor II (220491, 10 μM) was purchased from Merck Millipore, Darmstadt, Germany. Akt inhibitor IV (124011, 5 µM) was purchased from Cell Signaling, Beverly, MA, USA. Cells were treated with all drugs for 24 h except HU, which added for 72 h to induce prolonged replication stress.

### Antibodies

The following antibodies were obtained commercially: anti-histone H2AX (phospho-Ser139; GTX628789), anti-ATM (GTX70103), anti-Ku70 (GTX101820), anti-Ku80 (GTX109985), and anti-actin (AC-15; GTX26276) (GeneTex, Irvine, CA); anti-TFE3 (HPA023881), anti-acetylated-tubulin (T7451), anti-γ-tubulin (T5326), anti-α-tubulin (T9026) and anti-TTBK2 (HPA018113) (Sigma, St. Louis, MO); anti-pericentrin (ab4448), anti-ATM (phospho-Ser1981; ab81292) and anti-CP110 (ab99338) (Abcam, Cambridge, UK); polyclonal anti-IFT88 (13967-1-AP) and anti-ARL13B (17711-1-AP) (Proteintech, Chicago, IL); anti-DNA-PKcs (sc-9051), anti-DNA-PKcs (phospho-Thr2609; sc-101664), and anti-p53 (DO-1; sc-126) (Santa Cruz Biotech, CA, USA); anti-ULK1 (D8H5) rabbit mAb (#8054), anti-ULK1 (phospho-Ser757; #6888), anti-AMPK (#2532), anti-AMPK (phospho-Thr172; 40H9) rabbit mAb (#2535), anti-ATR (#2790), anti-ATR (phospho-Ser428; #2853), anti-LC3A/B (D3U4C) XP (#12741), anti-PCM1 (Q15; #5259), anti-Chk2 (#2662), anti-Chk2 (phospho-Thr68; #2661), anti-Akt (#9272), anti-Akt (phospho-Ser473; #4060), anti-p44/42 MAPK (phosphorylated Erk1/2; #9101), anti-p44/42 MAPK (Erk1/2; #9102), anti-p53 (phospho-Ser15; #9284), anti-Chk1 (2360), anti-Chk1 (phospho-Ser317; #12302), and anti-HSP70 (#4872) (Cell Signaling, Beverly, MA, USA); anti-CEP290 (A301-659A) (Bethyl Laboratories); anti-OFD1 (NBP1-89355) and anti-CEP164 (NBP1-81445) (Novus, Littleton, CO); and anti-ATG7 (EP1759Y; #04-1055) (Merck Millipore, Darmstadt, Germany).

### Immunofluorescence microscopy

Cells were grown on glass cover slips at 37 °C before fixation with ice-cold methanol at −20 °C for 6 min. After blocking with 5% BSA for 1 h, the cells were incubated with primary antibodies for 24 h at 4 °C. After extensive washing with PBS, the cells were incubated with fluorescein isothiocyanate- and Cy3-conjugated secondary antibodies (Invitrogen, Carlsbad, CA) for 1 h in the dark. The nuclei were stained simultaneously with 4′,6-diamidino-2-phenylindole (DAPI, 0.1 μg/ml). After extensive washing, the cover slips were mounted on glass slides in 50% glycerol. Fluorescent cells were examined with an Axio Imager M2 fluorescence microscope (Zeiss, Switzerland). Primary cilia were imaged with an Axio Imager M2 fluorescence microscope (Zeiss, Switzerland) and captured using ZEN pro software (Zeiss, Switzerland). Primary cilia images were created and the length of cilia were measured from z-stacks using add-on features of the ZEN pro software.

### RNA interference (RNAi)

DNA-PKcs, Ku70, ATM, Chk2, IFT88, and CEP164 were depleted in human RPE1 cells using annealed siRNAs with the following target sequences:

siDNA-PKcs: 5′-gggcgcuaaucguacugaa [dt] [dt]-3′ [16];

siKu70: 5′-gaugcccuuuacugaaaaa [dt] [dt]-3′ [16];

siATM: 5′-aacauacuacucaaagacauu [dt] [dt]-3′ [16];

siChk2: 5′-aagaaccugaggaccaagaac [dt] [dt]-3′ [16];

siIFT88: 5′-cgacuaagugccagacucauu [dt] [dt]-3′ [17];

siCEP164: 5′-caggugacauuuacuauuuca [dt] [dt]-3′ [18].

Scrambled siRNA (5′-gaucauacgugcgaucaga [dt] [dt]-3′) was purchased from Sigma (Sigma, St. Louis, MO).

For siRNA transfections, 10 μl Lipofectamine 2000 (Invitrogen, Carlsbad, CA) was mixed with 500 μl Opti-MEM (Life Technologies, Grand Island, NY) for 5 min, and 2 μl siRNA (100 μM) in 500 μl Opti-MEM was added to this mixture which was then incubated at room temperature for 20 min before being layered onto cells in 1 ml DMEM/F12 (100 nM working concentration). Cells were harvested for further experiments 72 h after transfection.

To generate recombinant lentivirus, plasmids expressing shRNA or envelope and packaging proteins were cotransfected into 293FT cells (Invitrogen, Carlsbad, CA), and virus was harvested according to the protocols provided by the Taiwan National RNAi Core Facility. The following short hairpin RNA (shRNA) sequences were introduced into the pLKO.1 vector:

pLKO.1-shluc (5′-ccuaagguuaagucgcccucg-3′) and pLKO.1-shATG7 (5′-gccugcugaggagcucuccau-3′).

Lentiviruses were collected from media of 293FT cells cotransfected with pLKO.1-derived plasmids and the packaging vectors pCMVdelR8.91 and pMD.G according to the protocols provided by the Taiwan National RNAi Core Facility.

### Generation of p53 knockout RPE1 cells

The p53 knockout RPE1 cells were kindly gifts from Dr. Won-Jing Wang. The stable p53 knockout RPE1 cells were established and published [19]. Briefly, RNA-guided targeting of p53 in RPE1 cells was performed through co-transfecting of Cas9 plasmid (Addgene Plasmid #41815) and guided-RNA targeting to p53 (5′-GGGCAGCTACGGTTTCCGTCTGG-3′). Cells were examined for the loss of p53 at 5, 6, or 7 days after transfection.

### Statistical analysis

All experiments were performed in at least three independent biological replicates and all results are expressed as the mean ± S.D. (the standard error) of three independent experiments, more than 100 cells were counted in each individual group. Differences between two groups were compared using unpaired two-tailed *t*-tests and ANOVA for multigroup comparisons, for which a *P* value of < 0.05 was statistically significant.

## Results

### Genotoxic drugs induce ciliogenesis

**Fig. 1:**
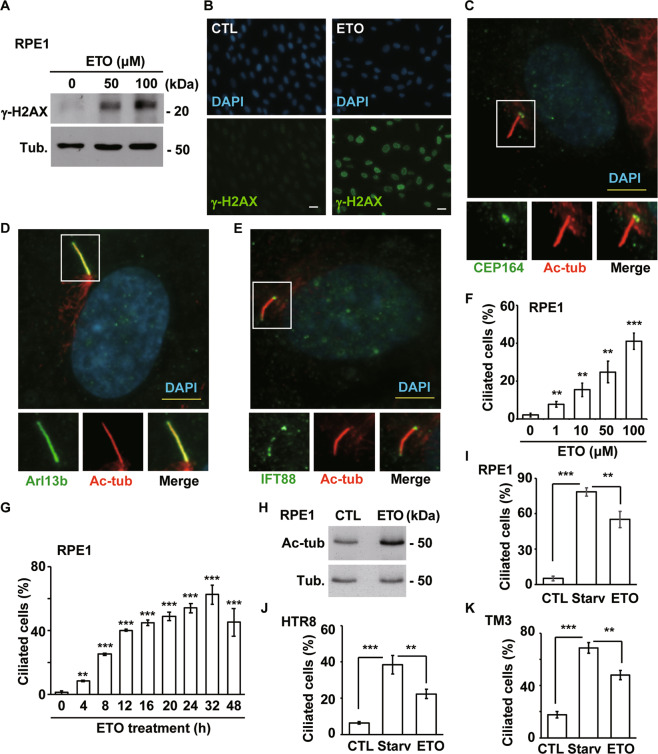
Genotoxic stress induces ciliogenesis. ETO treatment (50 and 100 μM, 24 h) induced DNA damage in RPE1 cells. **A** Extracts of RPE1 cells treated with or without ETO were analyzed by immunoblotting with antibodies against γ-H2AX and tubulin (Tub.). **B** γ-H2AX was detected by immunostaining with a specific antibody (green) in scramble control (CTL) or ETO-treated RPE1 cells. DNA was stained with DAPI (blue). Scale bar, 10 µm. ETO induced primary cilia formation in RPE1 cells. Double staining of ETO-treated cells with antibodies against (**C**) acetylated tubulin (Ac-tub, red) and CEP164 (green); **D** Ac-tub (red) and Arl13b (green); or **E** Ac-tub (red) and IFT88 (green). DNA was stained with DAPI (blue). Scale bar, 5 µm. **F** Quantitative results of the frequency of ciliated cells after treatment with different concentrations of ETO for 24 h. **G** Quantitative results of the frequency of ciliated cells after treatment with 100 µM ETO for different time periods. The results are presented as the mean ± SD of three independent experiments; more than 100 cells were counted in each individual group. **H** Ac-tub levels were increased in ETO-treated RPE1 cells. Extracts of RPE1 cells treated with or without ETO were analyzed by immunoblotting with antibodies against Ac-tub and Tub. Serum starvation (Starv) and ETO treatment induce ciliogenesis in RPE1 (**I**), HTR8 (**J**), and TM3 (**K**). **P < 0.01 and ***P < 0.001.

To investigate whether genotoxic stress induces formation of the primary cilium, the topoisomerase II inhibitor ETO, a known inducer of DNA double-strand breaks, was used. ETO treatment induced DNA damage, as shown by increased γ-H2AX levels (Fig. 1A, B). Then, primary cilia were examined in a human immortalized retina pigmented epithelium cell line (RPE1), an in vitro model for examining primary cilium formation [20]. Upon ETO treatment, acetylated tubulin, an axoneme marker, protruded from the mother centriole, as shown by CEP164 staining (Fig. 1C). To further confirm whether this acetylated tubulin signal contains an intact ciliary component rather than representing elongation of the mother centriole, other ciliary markers, including a known ciliary membrane protein (Arl13b) and an intraflagellar transporter (IFT88), were examined. Both Arl13b (Fig. 1D), and IFT88 (Fig. 1E), colocalized with acetylated tubulin, suggesting that these primary cilia had intact ciliary structure. Then, the cilia frequency (% of ciliated cells in a population) of RPE1 cells was counted. Upon ETO treatment, the population of ciliated cells increased in a dose- and time-dependent manner (Fig. 1F, G), and the abundance of acetylated tubulin also increased (Fig. 1H), suggesting that ETO induced primary cilia formation. Serum starvation induces ciliogenesis [21]. Despite ETO induced primary cilia formation, the population of ciliated cells induced by ETO was lower than that induced by starvation (Fig. 1I). During serum starvation, ciliogenesis begins with when tau tubulin kinase 2 (TTBK2) is recruited to the mother centriole, followed by phosphorylation of CP110, which caps the distal end of the centriole to prevent ciliogenesis [20]. Then, we examined whether ciliogenesis initiation events during serum starvation also occurred in ETO-treated RPE1 cells. TTBK2 recruitment to the mother centriole and removal of CP110 (Supplementary Fig. S1A, B), were also observed in ETO-treated RPE1 cells. Thus, ETO induces ciliogenesis by normal ciliogenesis initiation events in RPE1 cells.

Next, we examined whether ETO induces ciliogenesis in other cell lines. Human immortalized trophoblasts (HTR8) and mouse Leydig progenitor (TM3) cells grew primary cilia during serum starvation (Fig. 1J, K). Upon ETO treatment, these cell lines also showed the formation of primary cilia. As observed in RPE1 cells, more ciliated cells were observed under serum starvation in HTR8 and TM3 cells. These data suggest that, like serum starvation but not as efficiently, ETO induces primary cilia in all the tested cell lines. Several studies have shown that cancer cells do not grow cilia, but some recent studies have reported that some cancer cell lines, such as the human adenocarcinomic alveolar basal epithelial A549 cell line, are capable of growing primary cilia. These recent findings prompted us to ascertain whether A549 cells grow cilia in the presence of ETO. First, we tested whether A549 cells grew cilia under ciliogenic conditions, including those of serum starvation [18] and actin depolymerization [22]; both serum starvation (Fig. S2A) and actin depolymerization (Supplementary Fig. S2B) facilitated ciliogenesis in A549 cells. Next, we examined whether ETO induced ciliogenesis in A549 cells. Similar to the results in RPE1, TM3, and HTR8 cells, ETO treatment induced ciliogenesis in A549 cells (Supplementary Fig. S2C), and all ciliary components were detected in ETO-treated A549 cells, supporting the hypothesis that ETO induces primary cilia formation in A549 cells (Supplementary Fig. S2D–F). Centrosome amplification (cells with more than three centrosomes) has been observed in ETO-treated cancer cells, such as osteosarcoma cells [16]. However, we only observed promotion of ciliogenesis (Supplementary Fig. S2C), but not centrosome amplification (Supplementary Fig. S2G), in ETO-treated A549 cells. Thus, ETO induces primary cilia formation in immortalized normal cell lines (RPE1, TM3, and HTR8) and in the A549 cancer cell line.

To examine whether ETO-induced ciliogenesis is a general effect of genotoxic stress, rather than a specific response to ETO, other genotoxic stress inducers were evaluated. Neocarzinostatin (NCS), Hydroxyurea (HU), 7-hydroxy staurosporine (UCN-01), and cisplatin (CPT) induced ciliogenesis in RPE1 cells (Supplementary Fig. S3A–D). In addition, CPT treatment induced ciliogenesis in A549 cells (Supplementary Fig. S3E). Collectively, genotoxic stress triggers primary cilia formation.

### DNA-PK induces ciliogenesis

**Fig. 2:**
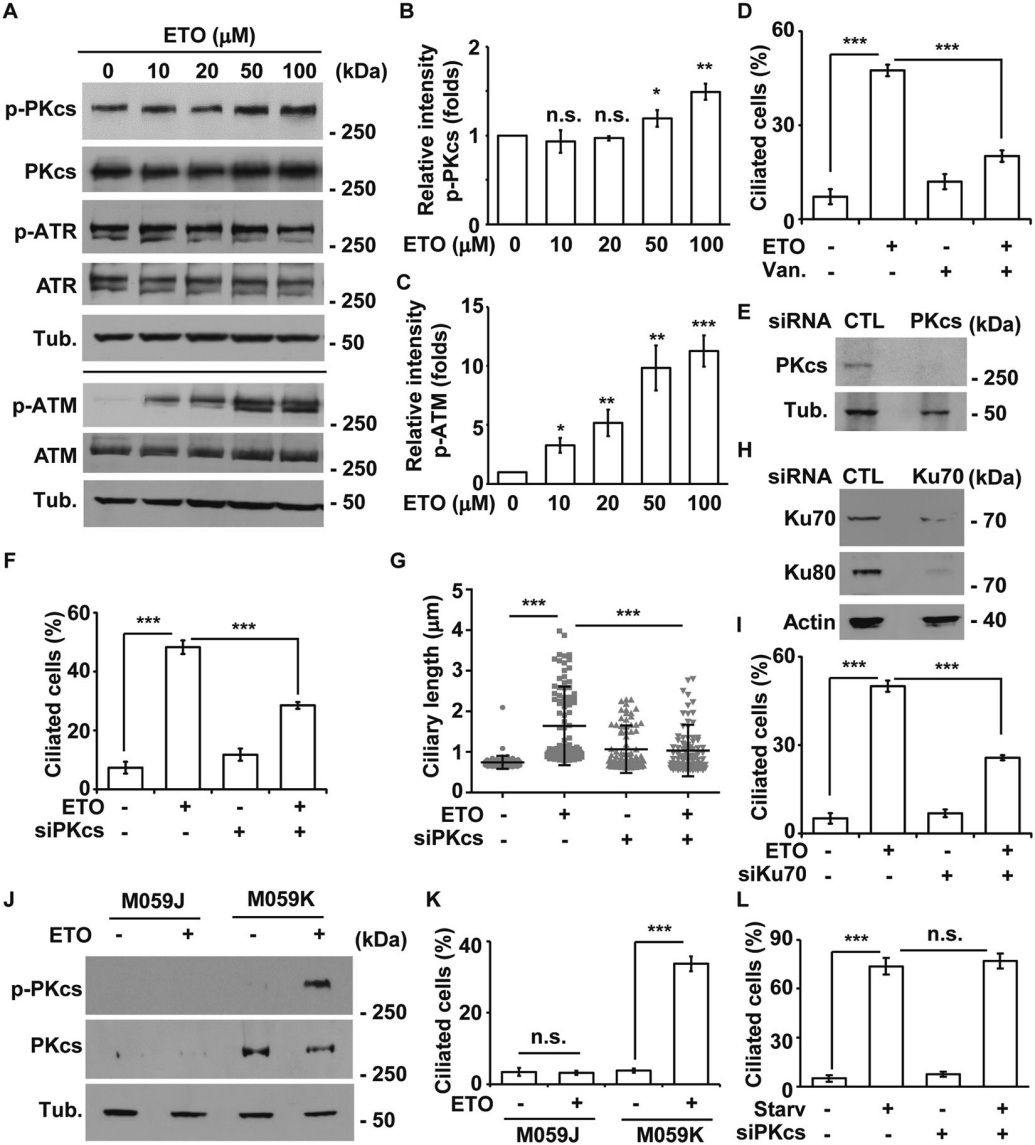
DNA-PK contributes to ciliogenesis in ETO-treated RPE1 cells. **A** ETO activated DNA-PK and ATM in RPE1 cells in a dose-dependent manner. Extracts of cells treated with different concentrations of ETO for 24 h were analyzed with antibodies against phosphorylated DNA-PKcs (p-PKcs), DNA-PKcs (PKcs), phosphorylated ATR (p-ATR), ATR, phosphorylated ATM (p-ATM), ATM, and tubulin (Tub). Quantitative results of relative intensity of p-PKcs/PKcs (**B**) and p-ATM/ATM (**C**) of **A**. All ETO-treated data were normalized to the data without ETO treatment. DNA-PK induced ciliogenesis upon ETO treatment. **D** Inhibition of DNA-PK by vanillin decreased ETO-induced ciliogenesis. Quantitative results of the frequency of ciliated RPE1 cells treated with 100 µM ETO for 24 h in the presence or absence of vanillin (Van.). siRNA-mediated depletion of DNA-PK decreased ETO-induced ciliogenesis. (E-G) Depletion of DNA-PKcs (siPKcs) diminished ETO-induced ciliogenesis. **E** DNA-PKcs was efficiently depleted. Extracts of cells transfected with siPKcs were analyzed by immunoblotting with antibodies against DNA-PKcs and tubulin. siPKcs reduced the frequency (**F**) and length (**G**) of cilia in ETO-treated RPE1 cells. Depletion of Ku70 (siKu70) decreased ETO-induced ciliogenesis. **H** Ku70 was efficiently depleted. Extracts of cells transfected with siKu70 were analyzed by immunoblotting with antibodies against Ku70, Ku80, and actin. **I** siKu70 reduced the frequency of ciliated ETO-treated RPE1 cells. The results are presented as the mean ± SD of three independent experiments; more than 100 cells were counted in each individual group. ETO-induced ciliogenesis was reduced in DNA-PK-deficient cells. **J** ETO activated DNA-PK in M059K but not in M059J cells. Extracts of M059J and M059K cells treated with ETO for 24 h were analyzed with antibodies against phosphorylated p-PKcs, PKcs, and tubulin (Tub.). **K** Quantitative results of the frequency of ciliated M059J and M059K cells treated with 100 µM ETO for 24 h. **L** Depletion of DNA-PKcs did not inhibit starvation-induced ciliogenesis. Quantitative results of the frequency of ciliated RPE1 cells under serum starvation for 24 h in control and DNA-PKcs-deficient (siPKcs) cells. *P < 0.05; **P < 0.01; ***P < 0.001; n.s. no significance.

Next, we examined whether the DNA damage response contributes to ciliogenesis. We first examined activation of the PI3KK family. Upon ETO treatment, ATM and DNA-PKcs, but not ATR, were activated in a dose-dependent manner in RPE1 and A549 cells (Fig. 2A–C and Supplementary Fig, S4A). To determine the effect of ATM activation on ciliogenesis, ATM activity was inhibited by caffeine, a pan ATM/ATR inhibitor, or Ku55933, an ATM-specific inhibitor. Neither caffeine nor Ku55933 inhibited ETO-induced ciliogenesis in RPE1 and A549 cells (Supplementary Fig. S4B–D). To further confirm this finding, ATM expression was knocked down by siRNA, which efficiently reduced ATM abundance upon transfection in RPE1 cells (Supplementary Fig. S4E). However, ATM depletion had no effect on ETO-induced ciliogenesis (Supplementary Fig. S4F). Thus, ATM activation does not contribute to primary cilia formation in ETO-treated cells.

We then examined whether DNA-PK activation contributes to ETO-induced ciliogenesis. Treatment of cells with the DNA-PK-specific inhibitor vanillin inhibited ETO-induced ciliogenesis (Fig. 2D). To examine whether DNA-PK-induced ciliogenesis is a general effect of genotoxic stress, HU and NCS were evaluated. HU and NCS activated DNA-PK and induced ciliogenesis in RPE1 cells (Supplementary Fig. S4G, H). Treatment of cells with vanillin inhibited HU- and NCS-induced ciliogenesis (Supplementary Fig. S4H), suggesting DNA-PK activation triggered primary cilia formation. Next, the catalytic subunit of DNA-PK (DNA-PKcs) was depleted by siRNA transfection (Fig. 2E), which decreased the frequency and length of ciliated cells (Figs. S4I and 2F, G). In addition, the regulatory subunits of DNA-PK, the Ku70-Ku80 heterodimers, were depleted by siRNA transfection. Depletion of Ku70 leads to reduced expression of Ku80 [23]. Consistent with published results, Ku80 expression was reduced in Ku70-deficient RPE1 cells (Fig. 2H), in which ETO-induced ciliogenesis was reduced (Fig. 2I), supporting DNA-PK induced primary cilia formation in ETO-treated cells. To further confirm this finding, glioblastoma cells, M059K and M059J, were used in our study, as M059K has normal DNA-PKcs while M059J fails to express the DNA-PKcs [24]. Upon ETO treatment, DNA-PKcs was expressed and activated in M059K cells but not in M059J cells (Fig. 2J). Consistently, ETO induced primary cilia formation only in M059K cells (Fig. 2K), suggesting DNA-PK induced ciliogenesis upon ETO treatment. Next, we examined whether DNA-PK participated in serum starvation-induced ciliogenesis. Serum starvation induced primary cilia formation; depletion of DNA-PKcs had no effect on starvation-induced ciliogenesis (Fig. 2L). Thus, DNA-PK induces primary cilia formation upon ETO treatment.

Next, the subcellular localization of activated DNA-PK, as shown by DNA-PKcs phosphorylation, was examined. Under normal conditions, phosphorylated DNA-PKcs was hardly detected in the nucleus, and a mild signal was asymmetrically detected in one centriole at interphase (Supplementary Fig. S5A). The phosphorylated DNA-PKcs signal increased dramatically in the centrosome when cells entered M phase (Supplementary Fig. S5B). ETO induced DNA-PKcs phosphorylation in the nucleus (Supplementary Fig. S5C), and phosphorylated DNA-PKcs was also detected in the basal body (mother centriole, 88.3 ± 5.7% of ciliated cells in a population) and daughter centriole (81.3 ± 5.5% of ciliated cells in a population), as shown by the colocolization of phosphorylated DNA-PKcs with p150^glued^ (marker protein of mother centriole, Supplementary Fig. S5D) and centrobin (marker protein of daughter centriole, Supplementary Fig. S5E). Thus, ETO induces DNA-PKcs phosphorylation in the nucleus, basal body (mother centriole), and daughter centriole.

### Activation of p53 induces ciliogenesis

**Fig. 3:**
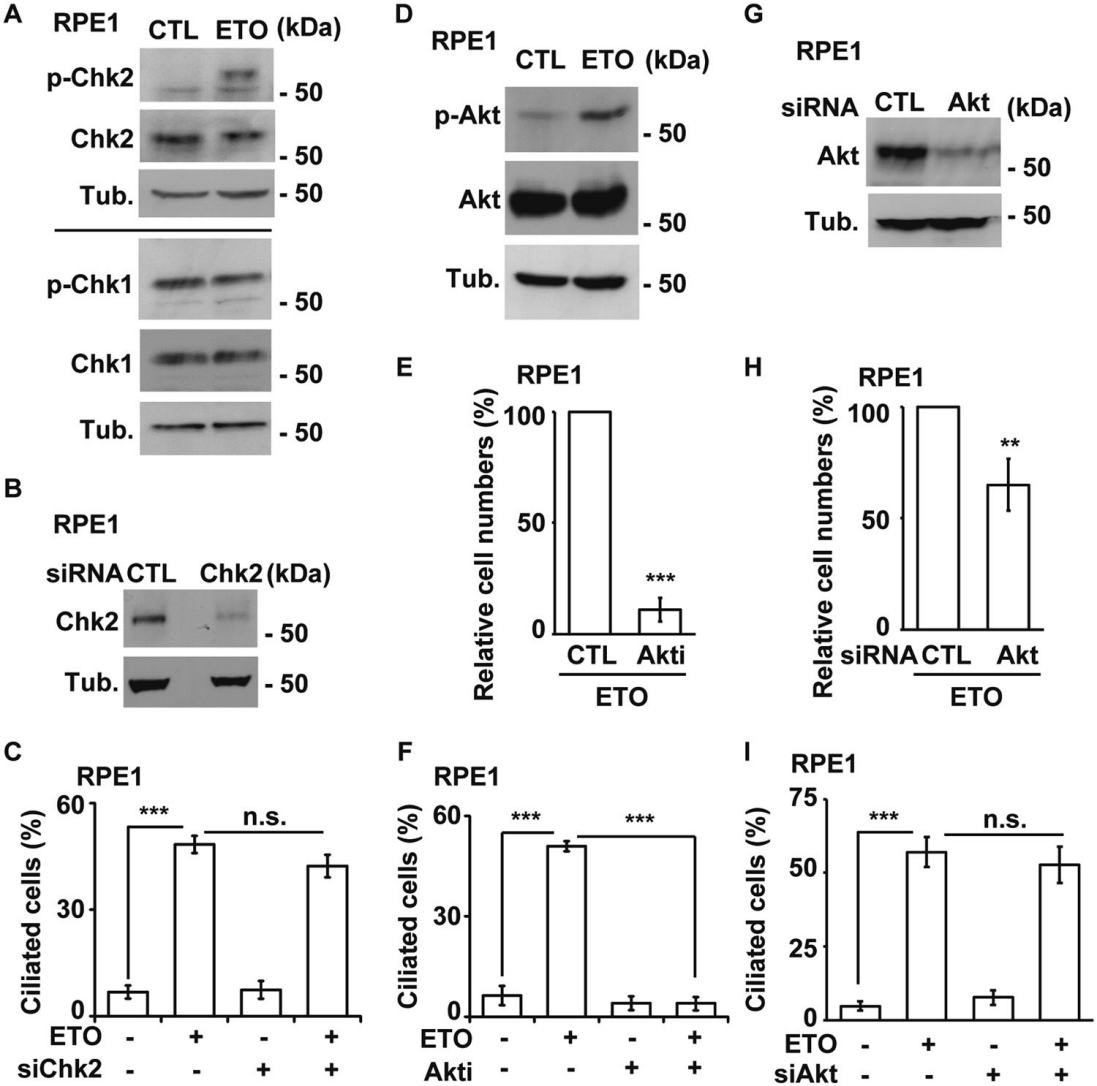
Akt activation does not promote ciliogenesis in RPE1 cells. Activated Chk2 did not contribute to ETO-induced ciliogenesis. **A** Chk2 was activated in ETO-treated RPE1 cells. Extracts of cells treated with ETO at 100 µM for 24 h were analyzed by immunoblotting with antibodies against phosphorylated Chk2 (p-Chk2), Chk2, phosphorylated Chk1 (p-Chk1), Chk1, and tubulin (Tub.). Depletion of Chk2 did not inhibit ETO-induced ciliogenesis. **B** Chk2 was depleted efficiently. Extracts of RPE1 cells transfected with siRNA against Chk2 was analyzed by immunoblotting with antibodies against Chk2 and tubulin (Tub.). **C** Quantitative results of frequency of ciliated RPE1 cells treated with 100 µM ETO for 24 h in control or Chk2-deficient cells. Activation of Akt did not induce ciliogenesis. **D** Akt was activated by ETO treatment. Extracts of cells treated with 100 µM ETO for 24 h were analyzed by immunoblotting with antibodies against phosphorylated Akt (p-Akt), Akt, and tubulin (Tub.). **E** inhibition of Akt led to robust cell death upon ETO treatment. Quantitative results of relative cell numbers after treatment with 100 µM ETO for 24 h in the presence or absence of Akt inhibitor IV (Akti, 5 µM). **F** Inactivation of Akt diminished ETO-induced ciliogenesis. Quantitative results of the frequency of ciliated cells after treatment with 100 µM ETO for 24 h in the presence or absence of Akt inhibitor IV (Akti, 5 µM). The results are presented as the mean ± SD of three independent experiments; more than 100 cells were counted in each individual group. **G** Akt was depleted efficiently. Extracts of RPE1 cells transfected with siRNA against Akt was analyzed by immunoblotting with antibodies against Akt and tubulin (Tub.). **H** Depletion of Akt had modest effect on cell death upon ETO treatment. Quantitative results of relative cell numbers after treatment with 100 µM ETO for 24 h in the control or Akt-deficient cells. **C** Quantitative results of frequency of ciliated RPE1 cells treated with 100 µM ETO for 24 h in control or Akt-deficient cells. **P < 0.01; ***P < 0.001; n.s. no significance.

The downstream effectors of PI3KK were further examined. We first evaluated checkpoint kinases. Chk2, but not Chk1, was activated by ETO treatment in RPE1 (Fig. 3A) and A549 (Supplementary Fig. S6A) cells. However, when Chk2 was efficiently depleted by siRNA transfection (Fig. 3B) or inactivated by the Chk2-specific inhibitor Chk2 inhibitor II, the frequency of ciliated cells was not affected (Fig. 3C and Supplementary Fig. S6B), suggesting that Chk2 activation does not contribute to ETO-induced ciliogenesis. We then investigated Akt activation, as DNA-PK activates Akt signaling to promote cell survival under stress [25]. ETO treatment activated Akt (Fig. 3D and Supplementary Fig. S6A). Inhibition of Akt induced robust cell death (about 90% of cell death, Fig. 3E) upon ETO treatment, consistent with the earlier report that inactivation of Akt induces apoptosis [25]. Examining the remaining live cells, we found that inhibition of Akt decreased ETO-induced ciliogenesis in both RPE1 and A549 cells (Fig. 3F and Supplementary Fig. S6C). As robust cell death was observed when Akt was inhibited in RPE1 and A549 cells, Akt expression was knocked down by siRNA, which efficiently reduced Akt abundance upon transfection in RPE1 cells (Fig. 3G). Depletion of Akt had modest effect on cell viability (about 40% of cell death, Fig. 3H), Examining the remaining live cells, however, Akt depletion had no effect on ETO-induced ciliogenesis (Fig. 3I). Thus, Akt activation does not contribute to primary cilia formation in ETO-treated cells.

**Fig. 4:**
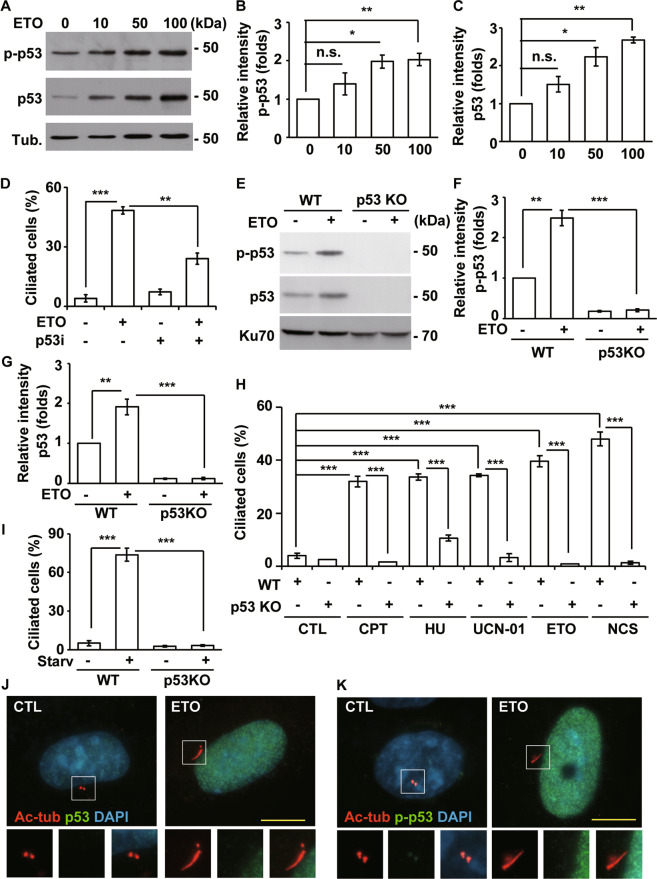
ETO induces p53 activation to promote ciliogenesis in RPE1 cells. **A** ETO activated p53 in RPE1 cells. Extracts of cells treated with different concentration of ETO for 24 h were analyzed with antibodies against phosphorylated p53 (p-p53), p53, and tubulin (Tub.). Quantitative results of relative intensity of p-p53/actin (**B**) and p53/actin (**C**) of **A**. All ETO-treated data were normalized to the data without ETO treatment. **D** Inhibition of p53 reduced ETO-induced ciliogenesis. Quantitative results of frequency of ciliated RPE1 cells treated with 100 µM ETO for 24 h in the presence or absence of p53 inhibitor, pifithrin-α. ETO-induced ciliogenesis were inhibited in p53 knockout RPE1 cells. **E** Extracts of wild-type (WT) or p53 knockout (p53 KO) cells treated with ETO for 24 h were analyzed with antibodies against p53 and Ku70. Quantitative results of relative intensity of p-p53/actin (**F**) and p53/actin (**G**) of E. **H** Knockout of p53 inhibited ciliogenesis. Quantitative results of frequency of ciliated RPE1 cells treated with cisplatin (CPT), hydroxyurea (HU), 7-Hydroxystaurosporine (UCN-01), ETO, and neocarzinostatin (NCS) for 24 h. **I** Knockout of p53 inhibited starvation (Starv)-induced ciliogenesis. Quantitative results of the frequency of ciliated RPE1 cells under serum starvation for 24 h in wild-type and p53 knockout cells. Total p53 (**J**) and phosphorylated p53 (**K**) did not localize to the cilia and centriole in ETO-treated RPE1 cells. Immunostaining of CTL- or ETO-treated RPE1 cells with antibodies against p53 (**J**) or p-p53 (**K**) and acetylated tubulin (Ac-tub). DNA was stained with DAPI (blue). Scale bar, 10 µm. *P < 0.05; **P < 0.01; ***P < 0.001; n.s. no significance.

We then examined p53, as it is a key downstream regulator of DNA-PK and plays an important role in genotoxic stress responses. Upon ETO treatment, total and phosphorylated p53 levels increased in a dose-dependent manner (Fig. 4A–C). Inhibition of p53 by the specific inhibitor pifithrin-α reduced ETO-induced ciliogenesis in RPE1 cells (Fig. 4D); thus, we speculated that p53 might be a crucial regulator. To further confirm the role of p53 in genotoxic stress-induced ciliogenesis, p53 was knocked out by CRISPR-Cas9 genome editing in RPE1 cells (p53 KO cells). The abundance of p53 was not detected in p53 KO cells (Fig. 4E). Upon ETO treatment, phosphorylated and total p53 levels increased in wild-type RPE1 cells but not in p53 KO cells (Fig. 4E–G), supporting p53 is depleted efficiently. Then, we examined whether genotoxic stress-induced ciliogenesis is regulated by p53. Treatment of wild-type RPE1 cells with CPT, HU, UCN-01, ETO, and NCS induced ciliogenesis; however, the frequency of ciliated cells and length of the cilia were reduced dramatically in p53 KO cells (Fig. 4H and Supplementary Fig. S7A), suggesting genotoxic stresses induce ciliogenesis via p53. Next, we examined whether p53 was activated by DNA-PK upon ETO treatment. ETO-activated p53 reduced significantly in DNA-PKcs-deficient cells implying DNA-PK activated p53 (Supplementary Fig. S7B–D). The effect of p53 on DNA-PK activation was also examined. Activation of DNA-PK was not reduced in the p53KO cells when compared with wild-type cells under ETO treatment, suggesting that p53 did not affect DNA-PK activation (Fig. S7E, F). We also examined whether p53 participated in serum starvation-induced ciliogenesis. Serum starvation induced primary cilia formation, and the population of ciliated cells was reduced dramatically in p53KO cells (Fig. 4I). Thus, p53 induces primary cilia formation under genotoxic stresses and serum starvation. The subcellular localizations of p53 and phosphorylated p53 were further examined. Under normal conditions, p53 (Fig. 4J) and phosphorylated p53 was hardly detected (Fig. 4K). ETO induced both p53 and phosphorylated p53 in the nucleus but not in the basal body (mother centriole) or daughter centriole. Thus, unlike the subcellular localization of activated DNA-PKcs, ETO induces abundances of p53 and phosphorylated p53 only in the nucleus. Collectively, genotoxic stress triggers primary cilia formation via p53.

### Autophagy induces ciliogenesis

**Fig. 5:**
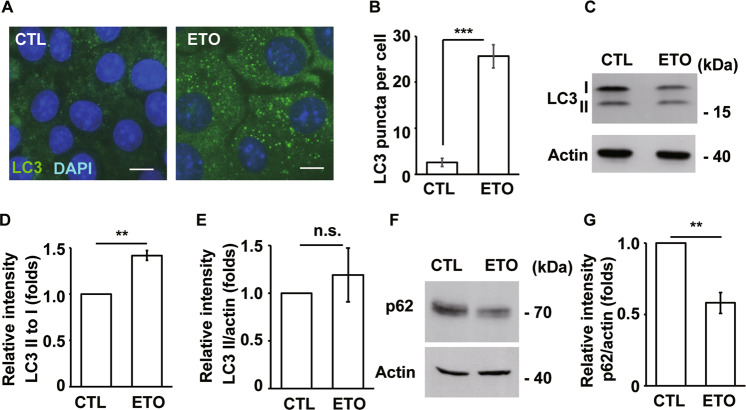
ETO treatment activates autophagy. **A** LC3 puncta were increased in ETO-treated RPE1 cells as shown by immunostaining with an antibody against LC3. DNA was stained with DAPI (blue). Scale bar, 10 µm. **B** Quantitative results of LC3 puncta per cell of RPE1 cells upon ETO treatment for 24 h. Increased LC3 II to I ratio in ETO-treated RPE1 cells. **C** Extracts of ETO-treated RPE1 cells were analyzed by immunoblotting with antibodies against LC3 and actin. Quantitative results of relative intensity of LC3 II/ I (**D**) and LC3 II/actin (**E**) of **C**. ETO-treated data were normalized to the data without ETO treatment. Reduced p62 abundance in ETO-treated RPE1 cells. **F** Extracts of ETO-treated RPE1 cells were analyzed by immunoblotting with antibodies against p62 and actin. **G** Quantitative results of relative intensity of p62 of **F**. **P < 0.01; ***P < 0.001; n.s. no significance.

Cellular stresses activate autophagy to maintain cell survival or induce apoptosis [12]. Autophagy also participates in ciliogenesis by degrading OFD1 during serum deprivation [13]. This finding prompted us to examine whether autophagy participates in ETO-induced ciliogenesis. First, we examined whether ETO treatment induces autophagy. The LC3 puncta were hardly detected in unstressed cells. However, upon ETO treatment, LC3 puncta increased throughout the cytoplasm in RPE1 (Fig. 5A, B) and A549 (Supplementary Fig. S8A, B) cells, suggesting that autophagy was affected. LC3 puncta might result from accelerated autophagic flux or reduced autophagic degradation; thus, LC3 was analyzed by immunoblotting. Upon ETO treatment, LC3 I levels were reduced in RPE1 (Fig. 5C) and A549 (Supplementary Fig. S8C) cells. In addition, LC3 II to I ratio increased upon ETO treatment (Fig. 5D); however, LC3 II to actin ratio was not significantly affected (Fig. 5E). Furthermore, the levels of p62 reduced significantly (Fig. 5F, G). The data imply that more LC3 I was converted to LC3 II, followed by lysosomal degradation. To further confirm this finding, the cells were treated with chloroquine (CQ), a well-established lysosomal inhibitor, and the conversion of LC3 II to I was examined. More LC3 II to I ratio was observed in ETO-treated or serum starvation-cultured cells than in control cells, supporting that more LC3 I was converted to LC3 II (Supplementary Fig. S8D). This result was further confirmed by cotreating RPE1 cells with ETO and Bafilomycin A1 (Baf.A1, an autophagic flux inhibitor) (Supplementary Fig. S8E). Thus, the data suggest that ETO induces autophagic flux. During serum deprivation, activated autophagic flux removes OFD1 from the centriolar satellite for ciliogenesis [13]. Therefore, we examined the abundance of OFD1 in the centriolar satellite. ETO treatment induced ciliogenesis, and OFD1 was displaced from the centriolar satellite (Supplementary Fig. S8F). However, genetic depletion of ATG7, DNA-PK, or p53 could not reverse displaced OFD1 phenotype upon ETO treatment (Supplementary Fig. S8G). The data show that ETO activates autophagic flux; however, OFD1 displacement is independent of autophagy and DNA-PK-p53 cascade upon ETO treatment.

**Fig. 6:**
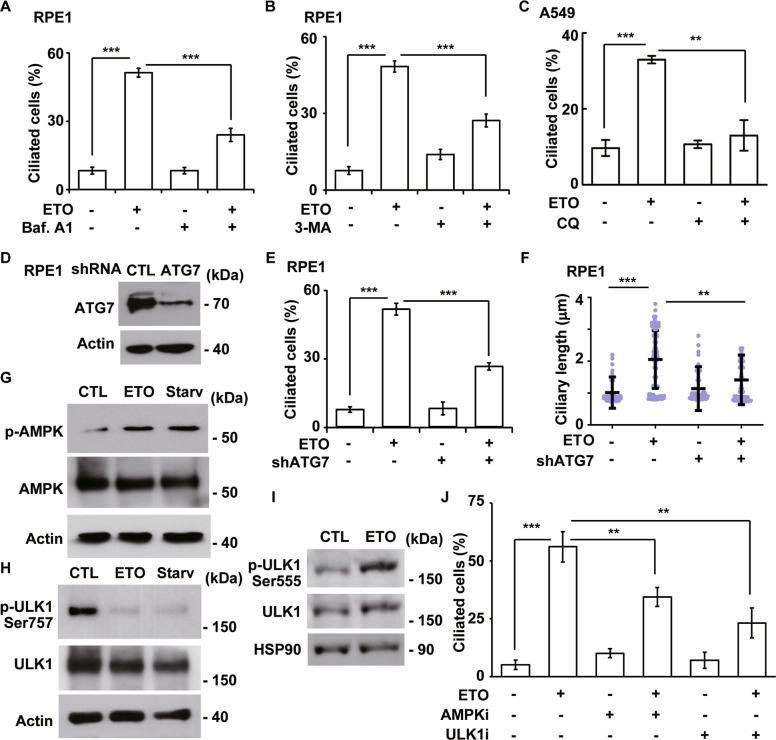
ETO-activated autophagy promotes ciliogenesis. ETO-induced autophagy facilitates ciliogenesis. Inhibition of lysosomal degradation by Baf.A1 (**A**) or autophagy initiation by 3-MA (**B**) reduced the frequency of ciliated RPE1 cells upon ETO treatment. **C** Inhibition of lysosomal degradation by CQ inhibited the frequency of ciliated A549 cells upon ETO treatment. Depletion of ATG7 by transfection of lentivirus containing shRNA against ATG7 (shATG7) inhibited ciliogenesis upon ETO treatment. **D** ATG7 was efficiently depleted. Extracts of RPE1 cells infected with shATG7 lentivirus were analyzed by immunoblotting with antibodies against ATG7 and actin. Quantitative results of the frequency (**E**) and length (**F**) of ciliated control and ATG7-deficient RPE1 cells in the absence or presence of ETO. **G**–**I** ETO treatment activated AMPK-ULK1 complex. Extracts of RPE1 cells treated with control (CTL), ETO, or serum starvation (Starv) for 24 h were analyzed with antibodies against phosphorylated AMPK (at Thr172), AMPK, phosphorylated ULK1 (at Ser757 or Ser555), actin, and HSP90. **J** Inactivation of AMPK and ULK1 reduced ETO-induced ciliogenesis. Quantitative results of the frequency of ciliated control, dorsomorphin- (5 μM; AMPKi), or SBI-0206965- (10 μM; ULK1i) treated RPE1 cells in the absence or presence of ETO. The results are presented as the mean ± SD of three independent experiments; more than 100 cells were counted in each individual group. **P < 0.01; ***P < 0.001.

Next, we examined whether ETO-induced autophagy contributes to ciliogenesis. Autophagic degradation was blocked by treating cells with Baf.A1, and the frequency of ciliated cells was counted. ETO induced ciliogenesis, and the frequency of ciliated cells was decreased in Baf.A1-treated RPE1 cells (Fig. 6A). This finding was further confirmed by treating cells with 3-Methyladenine (3-MA), an inhibitor of autophagy initiation (Fig. 6B). In A549 cells, treatment with CQ decreased ETO-induced ciliogenesis (Fig. 6C). To confirm that these results were not due to off-target effects of these drugs, a genomic approach was adopted. ATG7 expression was depleted by infecting RPE1 cells with lentivirus containing shRNA against ATG7 (Fig. 6D). Infection of lentivirus containing shRNA against luciferase (control) had no effect on ETO-induced ciliogenesis, but the frequency (Fig. 6E) and length (Fig. 6F) of ciliated cells were reduced in ATG7-deficient cells. Next, the involvement of AMPK-ULK1 complex was examined. Both serum starvation and ETO treatment activated AMPK and ULK1 as shown by increased phosphorylation of AMPK at Thr172 and of ULK1 at Ser555 (active form) and reduced phosphorylation of ULK1 at Ser757 (inactive form) (Fig. 6G–I). Inhibition of AMPK and ULK1 by selective inhibitors dorsomorphin and SBI-0206965, respectively, alleviated ETO-induced ciliogenesis (Fig. 6J), suggesting ETO-activated AMPK-ULK1 complex induced ciliogenesis. Thus, ETO-induced autophagy facilitates ciliogenesis.

**Fig. 7:**
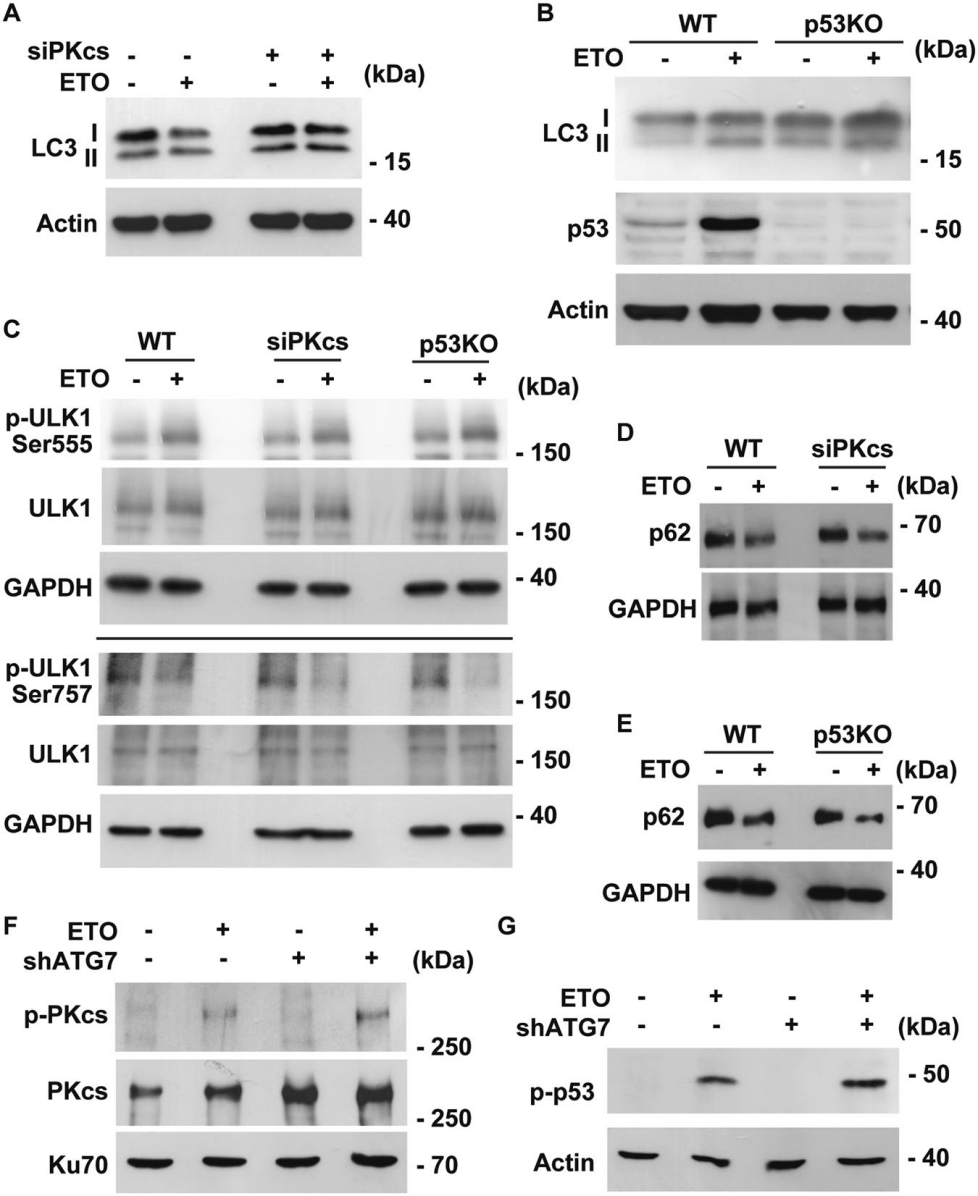
DNA-PK-p53 cascade does not affect autophagic flux upon ETO treatment. **A** Depletion of DNA-PKcs reduced LC3 II to I ratio. Extracts of control or DNA-PKcs-deficient (siPKcs) cells treated with ETO for 24 h were analyzed with antibodies against LC3 and actin. **B** LC3 II to I ratio was reduced in p53 knockout cells. Extracts of wild-type (WT) or p53 knockout (p53 KO) cells treated with ETO for 24 h were analyzed with antibodies against LC3, p53, and actin. DNA-PK-p53 cascade did not contribute to autophagic flux upon ETO treatment. Depletion of DNA-PKcs (siPKcs) or p53 (p53KO) did not affect ULK1 (**C**) activation or p62 degradation (**D**, **E**) in ETO-treated RPE1 cells. Extracts of RPE1 cells treated with ETO for 24 h were analyzed with antibodies against phosphorylated ULK1 (at Ser757 or Ser555), p62, and GAPDH. Knockdown of ATG7 did not inhibit DNA-PK (**F**) and p53 (**G**) activation. Extracts of control or ATG7-deficient (shATG7) cells treated with ETO for 24 h were analyzed with antibodies against phosphorylated PKcs (p-PKcs), PKcs, phosphorylated p53 (p-p53), Ku70, and actin. **E**–**H** ETO-activated AMPK-ULK1 complex induced ciliogenesis.

We then examined whether ETO-induced autophagy was regulated by DNA-PK-p53 cascade. ETO treatment increased LC3 II to I ratio in wild-type cells, and this was reduced in DNA-PKcs- or p53-deficient cells (Fig. 7A, B). To further strengthen the role of DNA-PK-p53 cascade over autophagy, activation of ULK1 and degradation of p62 was examined. Surprisingly, depletion of DNA-PKcs or p53 affect neither ULK1 activation nor p62 degradation (Fig. 7C–E). The data imply that ETO-activated DNA-PK-p53 cascade affects LC3 lipidation but not the flux of autophagy. DNA damage activates transcription factor E3 (TFE3) [26] and nuclear translocation of TFE3 promotes autophagy [27]. We then checked whether activation of TFE3 (nuclear translocation of TFE3) was regulated by DNA-PK-p53 cascade. ETO treatment facilitated nuclear translocation of TFE3 (Supplementary Fig. S9A); however, genomic depletion of DNA-PKcs or p53 did not affect TFE3 activation (Supplementary Fig. S9B, C), suggesting TFE3 was not regulated by DNA-PK-p53 cascade upon ETO treatment. The effect of autophagy on DNA-PK-p53 cascade was further examined. Depletion of ATG7 affected neither DNA-PK (Fig. 7F) nor p53 (Fig. 7G) activation upon ETO treatment. The data suggest that DNA-PK-p53 cascade and autophagy are independent pathways triggered by genotoxic stress.

### Primary cilia maintain DNA damage response

**Fig. 8:**
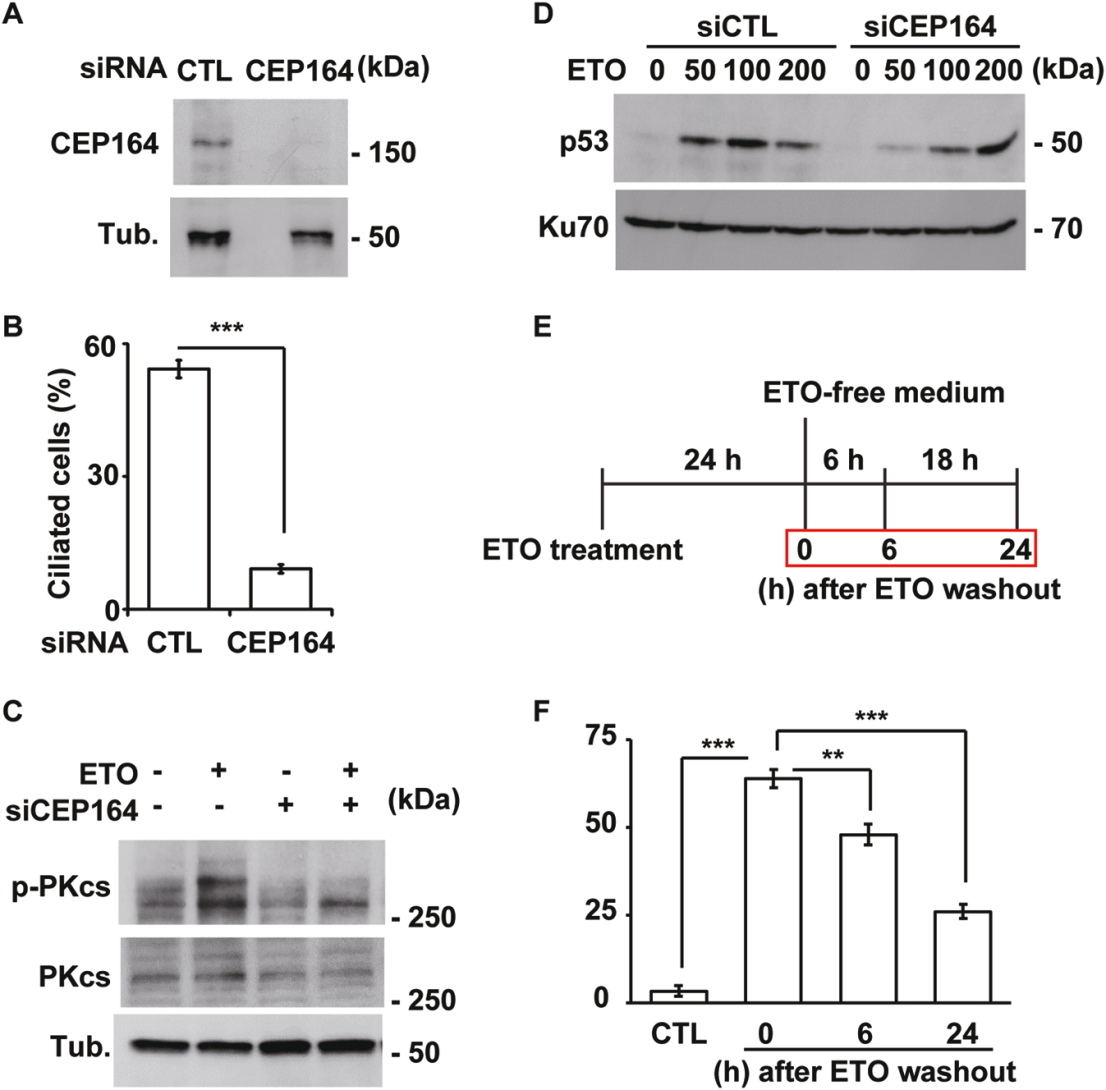
The primary cilium maintains the ETO-induced DNA damage response. Inhibition of ciliogenesis decreased ETO-induced DNA-PK activation. **A** Depletion of CEP164 inhibited ETO-induced DNA-PK activation. Extracts of RPE1 cells transfected with siRNA against CEP164 in the presence of ETO were analyzed by immunoblotting with antibodies against CEP164 and tubulin (Tub.). **B** Quantitative results of the frequency of ciliated CEP164-deficient RPE1 cells in the presence of ETO. The results are presented as the mean ± SD of three independent experiments; more than 100 cells were counted in each individual group. **C** Extracts of RPE1 cells transfected with siRNA against CEP164 were analyzed by immunoblotting with antibodies against phosphorylated DNA-PKcs (p-PKcs), DNA-PKcs (PKcs), and tubulin (Tub.). **D** Depletion of CEP164 inhibited ETO-induced p53 activation. Extracts of RPE1 cells transfected with siRNA against CEP164 in the presence of different concentrations of ETO were analyzed by immunoblotting with antibodies against p53 and Ku70. Removal of ETO reduced ETO-induced ciliogenesis in a time dependent manner. **E** Schematic representation of the experimental procedure used for **F**. Cells were treated with ETO for 24 h followed by culturing in ETO-free medium for additional 6 or 24 h before harvesting. **F** Quantitative results of the frequency of ETO-treated ciliated RPE1 cells after ETO washout. The results are presented as the mean ± SD of three independent experiments; more than 100 cells were counted in each individual group. **P < 0.01; ***P < 0.001.

Some centrosomal and ciliary proteins contribute to nuclear events. We thus ascertained whether ETO-induced primary cilia play a role in the DNA damage response. IFT88 and CEP164 are required for ciliogenesis; thus, the effects of primary cilia on the DNA damage response were examined in CEP164- and IFT88-deficient cells. CEP164 expression was efficiently depleted by siRNA transfection (Fig. 8A), which reduced the frequency of ciliated cells (Fig. 8B). Moreover, ETO induced DNA-PKcs phosphorylation, an indicator of DNA-PK activation, but this phosphorylation was reduced in CEP164-deficient RPE1 cells (Fig. 8C). This phenotype was further confirmed in IFT88-deficient cells (Supplementary Fig. S10A–C). The data suggest that primary cilia maintain DNA-PK activation. We then determined the effect of primary cilia on p53 activation. Similar to the results for DNA-PK, p53 activation by ETO treatment was reduced in CEP164-deficient cells (Fig. 8D). However, p53 was not reduced when cells were treated with ETO at lethal dose (200 μM). These data suggest that ETO-induced primary cilia maintain DNA-PK and p53 activation.

We next tested whether removal of genotoxic stress reduced ciliogenesis. RPE1 cells were treated with ETO for 24 h for ciliogenesis followed by washing with PBS and cultured in ETO-free medium for 6 and 24 h (Fig. 8E). Six hours after ETO washout, the percentage of ciliated cells reduced and more deciliated cells were observed 24 h after ETO washout (Fig. 8F). Thus, genotoxic stress sustains primary cilia formation.

## Discussion

Here, we showed that genotoxic stress facilitated primary cilium formation via the DNA-PK-p53 cascade and autophagy. Interestingly, DNA-PK-p53 cascade did not activate autophagy and vice versa. Moreover, stress-induced ciliogenesis was crucial for maintaining the DNA damage response (Fig. S11). Thus, our study unraveled the interplay among genotoxic stress, the primary cilium, and the DNA damage response.

Serum deprivation and genotoxic stress induced primary cilia formation in RPE1 cells. Depletion of DNA-PKcs inhibited ciliogenesis induced by genotoxic stress but not by serum deprivation (Fig. 2F, L). However, either genotoxic stress- or serum deprivation-induced ciliogenesis reduced significantly in p53 KO cells (Fig. 4H, I). Thus, DNA-PK-p53 cascade contributed to genotoxic stress-induced ciliogenesis and only p53 engaged in starvation-induced ciliogenesis, implying different signaling cascade are activated for ciliogenesis upon different cellular stresses. How does DNA-PK-p53 cascade regulate primary ciliogenesis remains unclear. Genotoxic stresses activate DNA-PK-p53 cascade for primary cilia formation. Activated DNA-PKcs localized to both nucleus and centrioles, p53 was only detected in the nucleus. We speculated that DNA-PK regulates ciliogenesis might via its nuclear and centriolar functions. p53 is a transcription factor that regulates several gene expression for regulating cell cycle progression. Interestingly, a study demonstrates a novel role of p53 in promoting differentiation of airway epithelial progenitors with motile cilia [28]. Thus, we propose that, in the nucleus, DNA-PK activates p53 thus inducing gene expressions for ciliogenesis. Activated p53 was not detected in the centrioles. Thus, in the centriole, DNA-PK might participate in orchestrating primary cilia by forming complex with other centriolar proteins or phosphorylating distinct signaling cascade for inducing or maintaining ciliogenesis. The hypothesis still needs to be further deciphered in the future.

Here we show that genotoxic stresses induce ciliogenesis; whereas disruption of primary cilia formation reduces DNA damage signaling. It remains unclear how cytoplasmic cilia affect nuclear signals. The role of primary cilia in the maintenance of DNA damage response is supported by previous finds that centrosomes contain several components participate in DNA damage responses [29]. Besides, several cilia-related proteins participate in DNA damage response. For example, CEP164 forms a complex with both ATM and ATR and is phosphorylated by these two kinases. This process is important for activating other DNA damage response components [30]. Interestingly, depletion of KIF3A reduces ciliogenesis and loss of cell cycle arrest in response to DNA damage due to reduced p53 activation [31]. Consistently, we found that disruption of ciliogenesis led to reduced DNA-PK and p53 activation. DNA-PK was found both in the nucleus and centrioles, we thus speculate that the shuttling of DNA-PK between nucleus and centrioles might play a role in maintaining DNA damage response. However, this hypothesis still needs to be further examined.

p53 plays roles in regulating autophagy [32]. In the nucleus, p53 induces Sestrin1 and Sestrin2 to activate the AMPK, thereby inhibiting mTOR [33, 34]. In the cytoplasm, p53-mediated proapoptotic factors such as p53-inducible BH3-only protein (PUMA) also contribute to activation of autophagy [35]. Our data showed that genotoxic stresses induced DNA-PK-p53 cascade. However, genomic depletion of DNA-PKcs or p53 did not affect ULK1 or TFE3 activation and p62 degradation, only LC3 lipidation was affected. We thus speculate that DNA-PK-p53 cascade and autophagy are independent pathways triggered by genotoxic stress but may still have some crosstalk. The detail interplay among DNA damage response and autophagy still need to be elucidated in the future.

In summary, we show that the DNA-PK-p53 cascade and autophagy induce primary cilia formation to maintain the DNA damage response under genotoxic stress. Thus, our study deciphered the molecular mechanism by which genotoxic stress induces primary cilium formation and uncovered the novel function of the primary cilium in maintaining the DNA damage response.

## Supplementary information

Supplementary data

Supplementary figure 1

Supplementary figure 2

Supplementary figure 3

Supplementary figure 4

Supplementary figure 5

Supplementary figure 6

Supplementary figure 7

Supplementary figure 8

Supplementary figure 9

Supplementary figure 10

Supplementary figure 11
