# Supplementary material for: Genotoxic stress-activated DNA-PK-p53 cascade and autophagy cooperatively induce ciliogenesis to maintain the DNA damage response
Source: Cell Death Differ. 2021 Jan 18;28(6):1865–79. doi: 10.1038/s41418-020-00713-8 (PMC8184926; doi:10.1038/s41418-020-00713-8)
Supplement: Supplementary file 1 — Supplementary data [file 41418_2020_713_MOESM1_ESM.docx]

**Supplementary figures**

**Supplementary figure 1.**


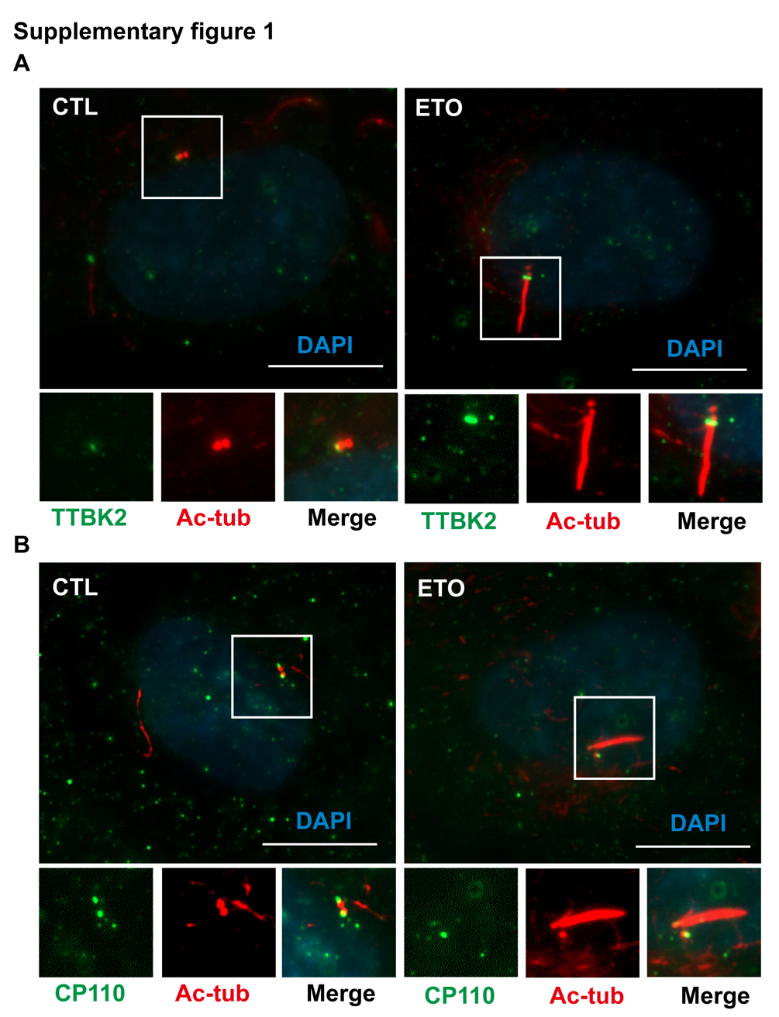


**Supplementary figure 1. Etoposide induces ciliogenesis via CP110 displacement.**

(A) TTBK2 was recruited to the base of primary cilium. Double staining of RPE1 cells in the absence (control, CTL; left panel) or presence of etoposide (ETO; right panel) with antibodies against TTBK2 and acetylated tubulin (Ac-tub). (B) CP110 was removed from the mother centriole. Double staining of RPE1 cells in the absence (control, CTL; left panel) or presence of etoposide (ETO; right panel) with antibodies against CP110 and acetylated tubulin (Ac-tub). DNA was stained with DAPI. Scale bars, 5 µm.

**Supplementary figure 2.**


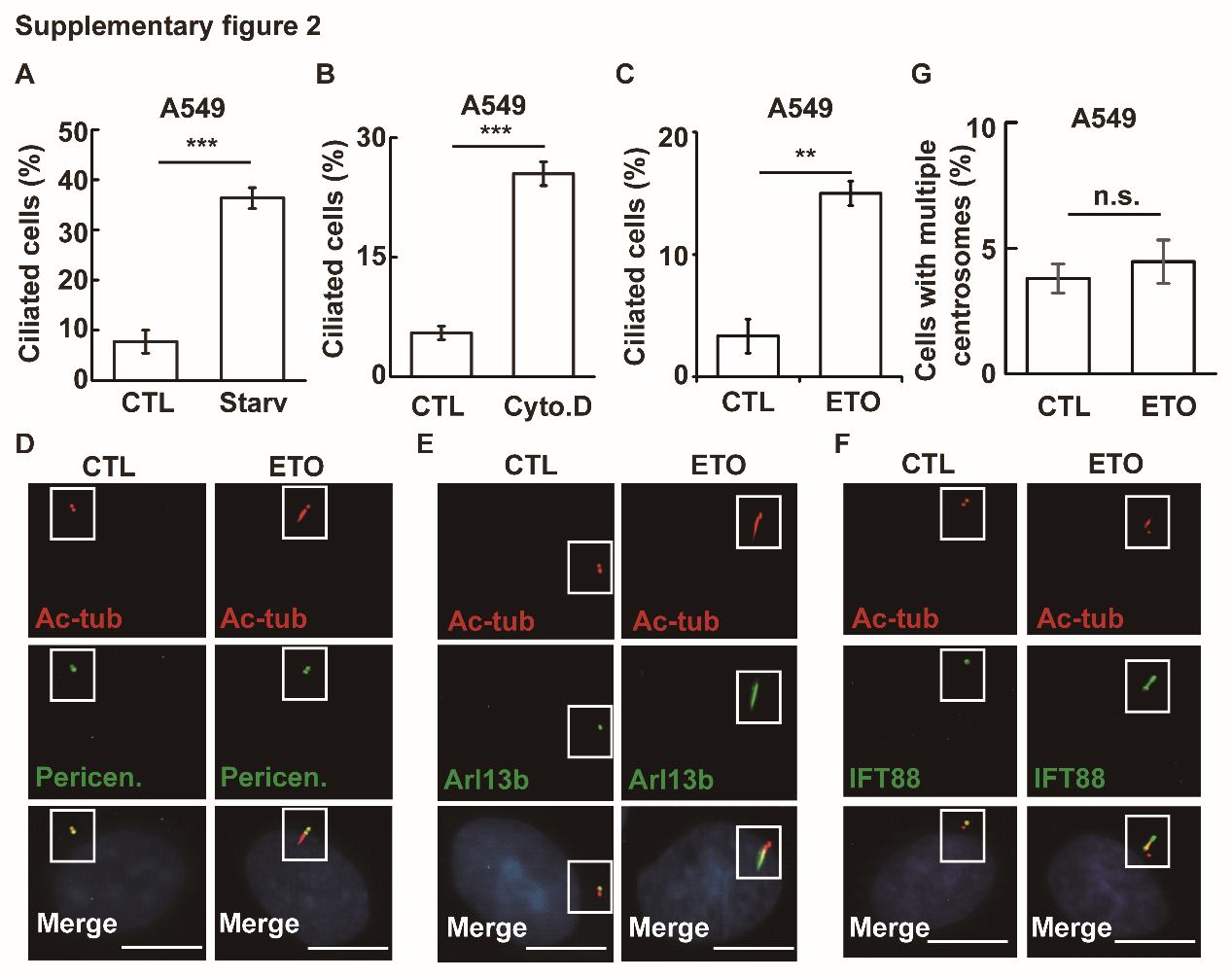


**Supplementary figure 2. Genotoxic stress induces ciliogenesis in several cell lines.**

(A-C) A549 cells could grow primary cilium under (A) serum starvation (Starv.), (B) actin depolymerization by cytochalasin D (Cyto. D) treatment, and (C) ETO treatment. (D-F) ETO-induced primary cilium contained essential ciliary components. Double staining of A549 cells in the absence (left panel) or presence of ETO (right panel) with antibodies against acetylated tubulin (Ac-tub) and (D) pericentrin (Pericen.), (E) Arl13b, or (F) IFT88. DNA was stained with DAPI. Scale bars, 5 µm. (G) ETO treatment did not induce multiple centrosome (cells with more than three centrosomes) formation. These results are mean +/- SD of three independent experiments; more than 100 cells were counted in each individual group. ** P<0.01; *** P<0.001; n.s. no significance.

**Supplementary figure 3.**


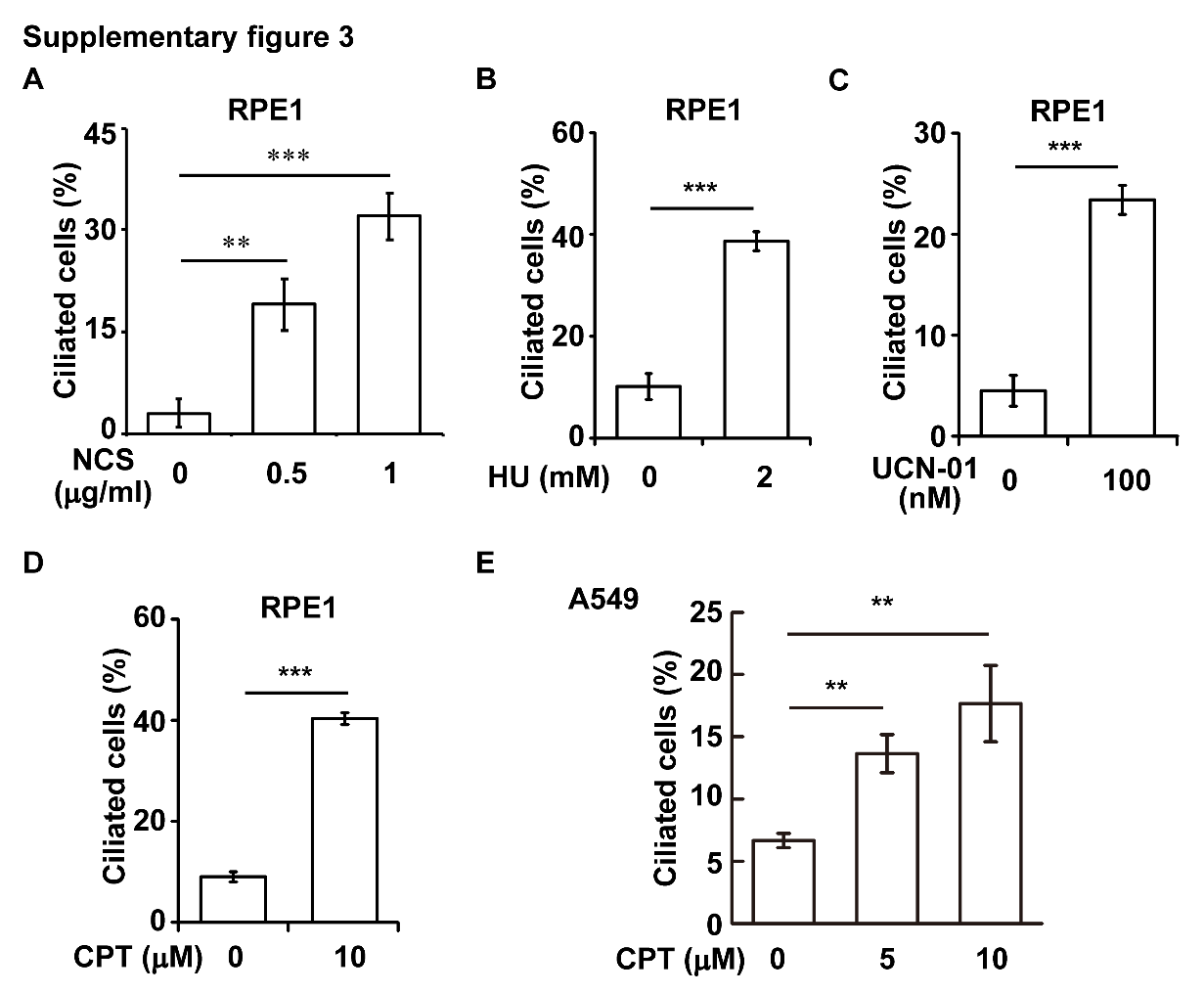


**Supplementary figure 3. Genotoxic stresses induce ciliogenesis.**

(A-E) Quantitative results of the frequency of ciliated cells when cells were treated with (A) neocarzinostatin (NCS, 24 h), (B) hydroxyurea (HU, 72 h), (C) UCN-01 (24 h), and (D) cisplatin (CPT, 24 h) in RPE1 cells, or (E) cisplatin treatment in A549 cells for 24 h. These results are mean +/- SD of three independent experiments; more than 100 cells were counted in each individual group. ** P<0.01; *** P<0.001.

**Supplementary figure 4.**


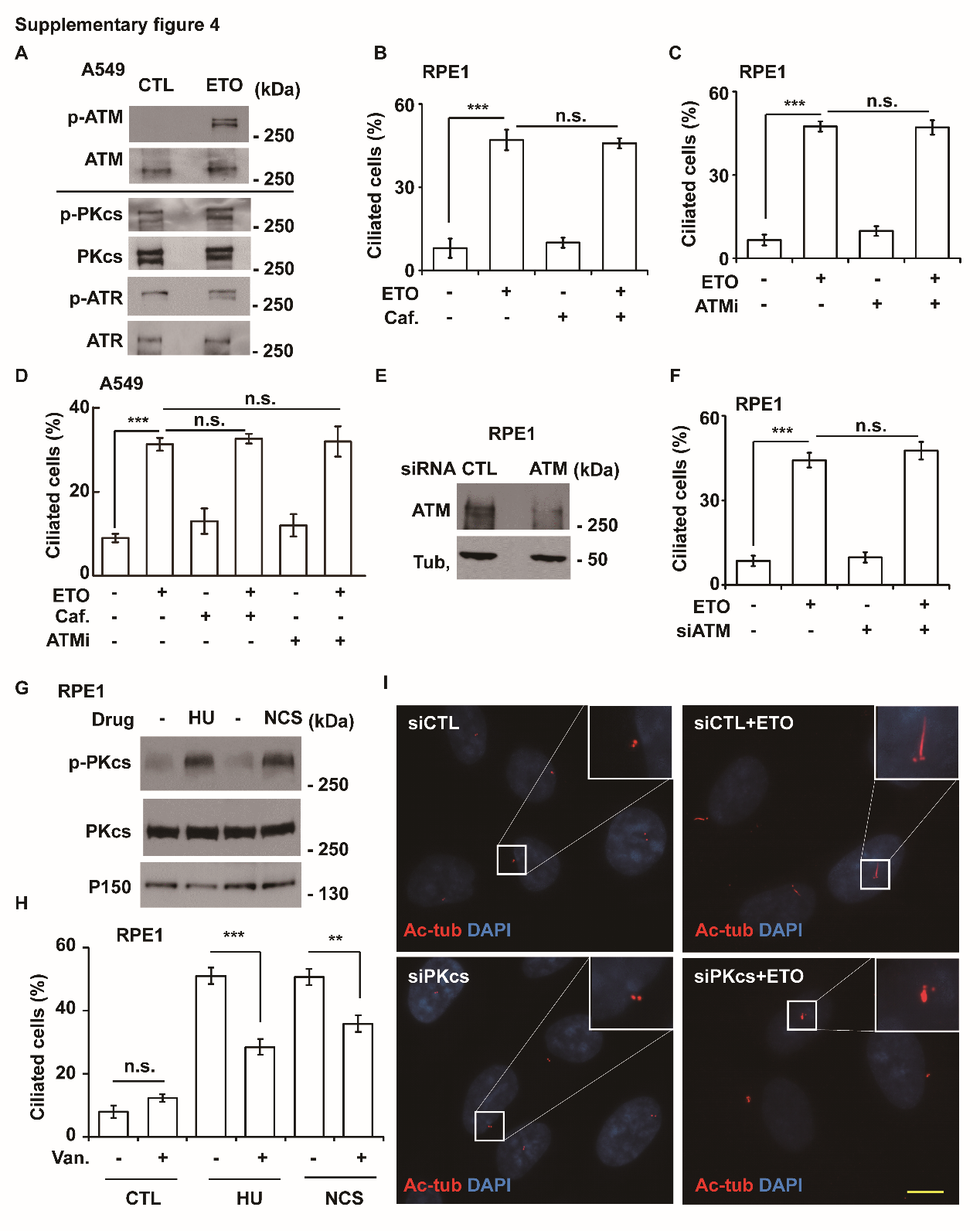


**Supplementary figure 4. Etoposide-induced ATM dose not induce ciliogenesis.**

(A) ETO activated DNA-PK and ATM in A549 cells. Extracts of cells treated with ETO at 100 µM for 24 h were analyzed by immunoblotting with antibodies against phosphorylated DNA-PKcs (p-PKcs), DNA-PKcs (PKcs), phosphorylated ATR (p-ATR), ATR, phosphorylated ATM (p-ATM), and ATM. (B-D) Inhibition of ATM did not affect ETO-induced ciliogenesis. Quantitative results of frequency of ciliated RPE1 (B-C) or A549 (D) cells treated with 100 µM ETO for 24 h in the presence or absence of (B and D) caffeine (Caf.), or (C and D) ATM inhibitor Ku55933 (ATMi). These results are mean +/- SD of three independent experiments; more than 100 cells were counted in each individual group. (E-F) Depletion of ATM did not affect ETO-induced ciliogenesis. (E) ATM was depleted efficiently. Extracts of siRNA against ATM were analyzed by immunoblotting with antibodies against ATM and tubulin (Tub.). Quantitative results of frequency of ciliated RPE1 cells treated with 100 µM ETO for 24 h when ATM is depleted (F). (G-H) ETO activated DNA-PK upon hydroxyurea (HU) and neocarzinostatin (NCS). (G) Extracts of cells treated with HU and NCS for 24 h were analyzed with antibodies against phosphorylated DNA-PKcs (p-PKcs), DNA-PKcs (PKcs), and P150. (C-H) ETO induced primary cilia formation in RPE1 cells. (I) Primary cilia were shown by immunostaining with antibodies against acetylated tubulin (Ac-tub, red) in control (CTL), ETO treated, or DNA-PKcs-deficient (siPKcs) cells. DNA was stained with DAPI (blue). Scale bar, 5 µm. ** P<0.01; *** P<0.001; n.s. no significance.

**Supplementary figure 5.**


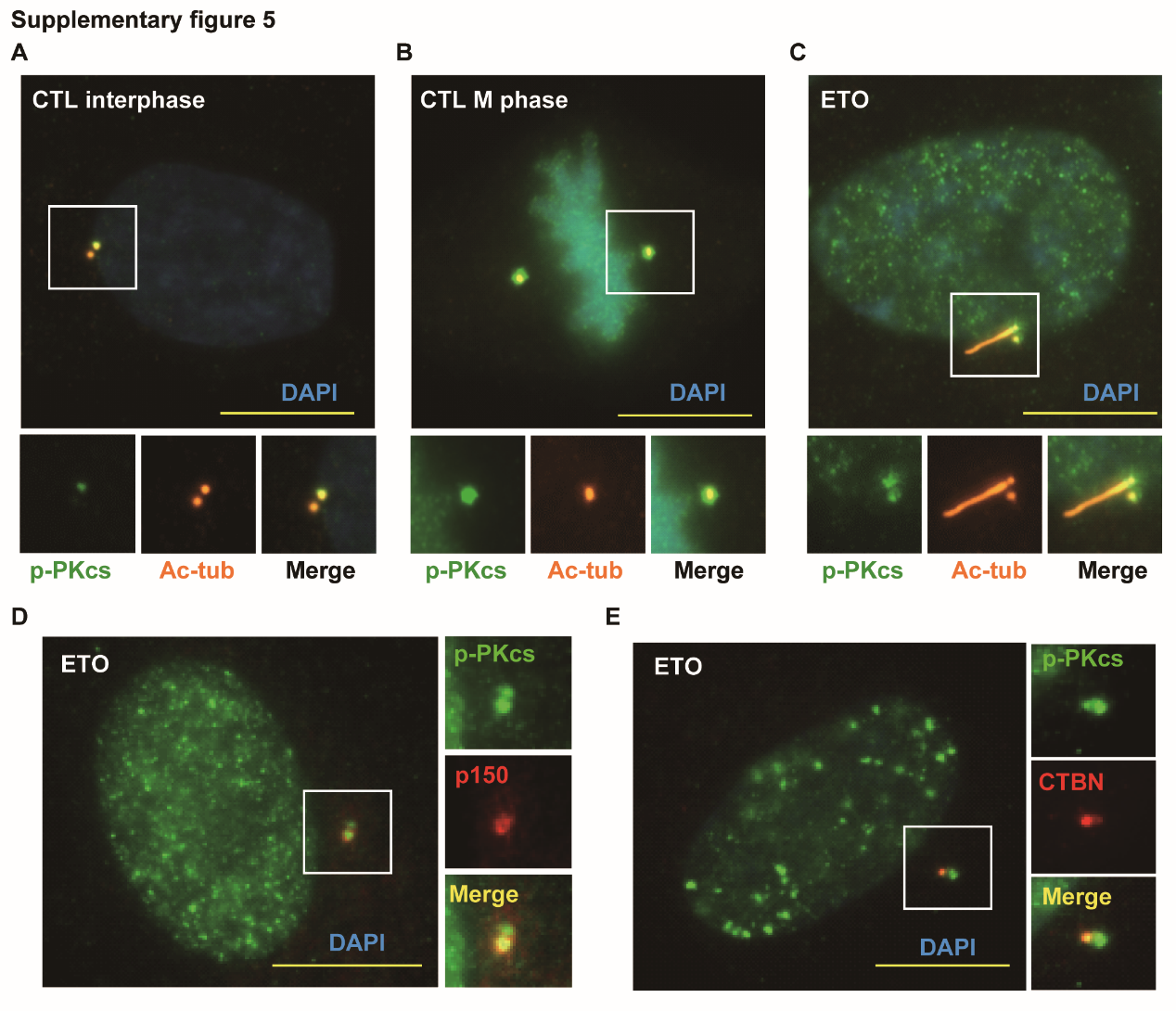


**Supplementary figure 5. Phosphorylated DNA-PKcs localizes to the nucleus, basal body (mother centriole), and daughter centriole upon ETO treatment.**

(A-C) Subcellular localization of phosphorylated DNA-PKcs (p-PKcs) at interphase (A) and M phase (B) in control (CTL) and ETO-treated (C) RPE1 cells. Immunostaining of CTL- or ETO-treated RPE1 cells with antibodies against phosphorylated DNA-PKcs (p-PKcs) and acetylated tubulin (Ac-tub). (D-E) Phosphorylated DNA-PKcs colocalized with mother (D) and daughter (E) centrioles. Mother centriole marker protein: p150glued (p150). Daughter centriole marker protein: centrobin (CTBN). DNA was stained with DAPI (blue). Scale bar, 10 µm.

**Supplementary figure 6.**


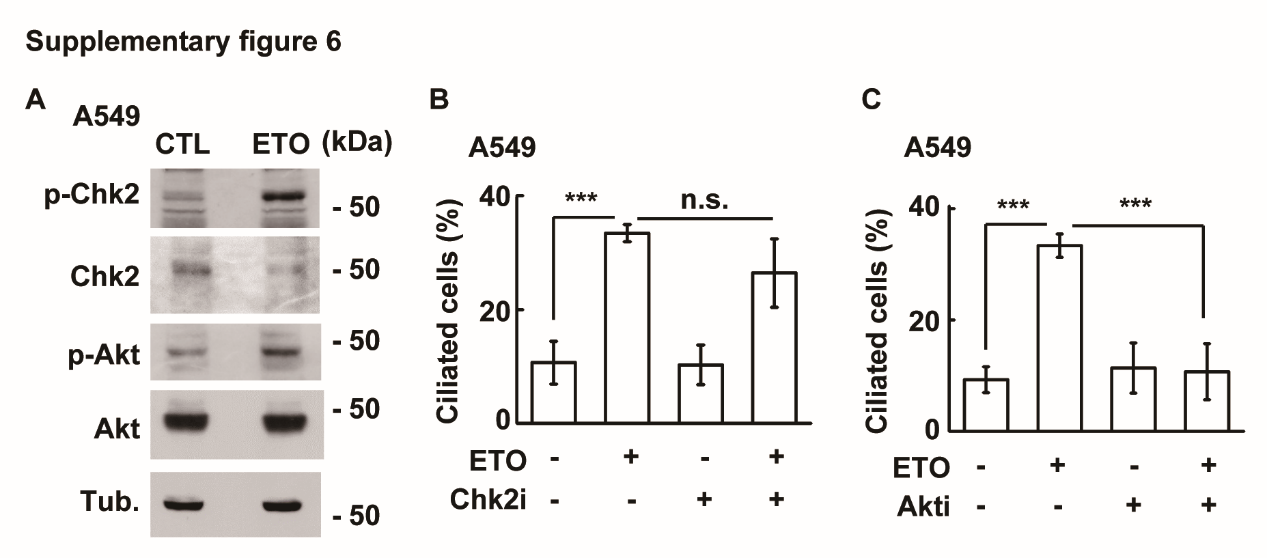


**Supplementary figure 6. Akt activation induces ciliogenesis upon etoposide treatment in A549 cells.**

(A-B) Activated Chk2 did not contribute to ETO-induced ciliogenesis. (A) Chk2 was activated in A549 cell lines. Extracts of cells treated with ETO at 100 µM for 24 h were analyzed by immunoblotting with antibodies against phosphorylated Chk2 (p-Chk2), Chk2, phosphorylated Akt (p-Akt), Akt, and tubulin (Tub.). (B) Inactivation of Chk2 did not inhibit ETO-induced ciliogenesis. Quantitative results of ciliated A549 cells treated with ETO in the presence or absence of Chk2 inhibitor (Chk2i). (C) Inactivation of Akt inhibited ETO-induced ciliogenesis. Quantitative results of ciliated A549 cells treated with ETO in the presence or absence of Akt inhibitor (Akti). These results are mean +/- SD of three independent experiments; more than 100 cells were counted in each individual group. *** P<0.001 and n.s. no significance.

**Supplementary figure 7.**


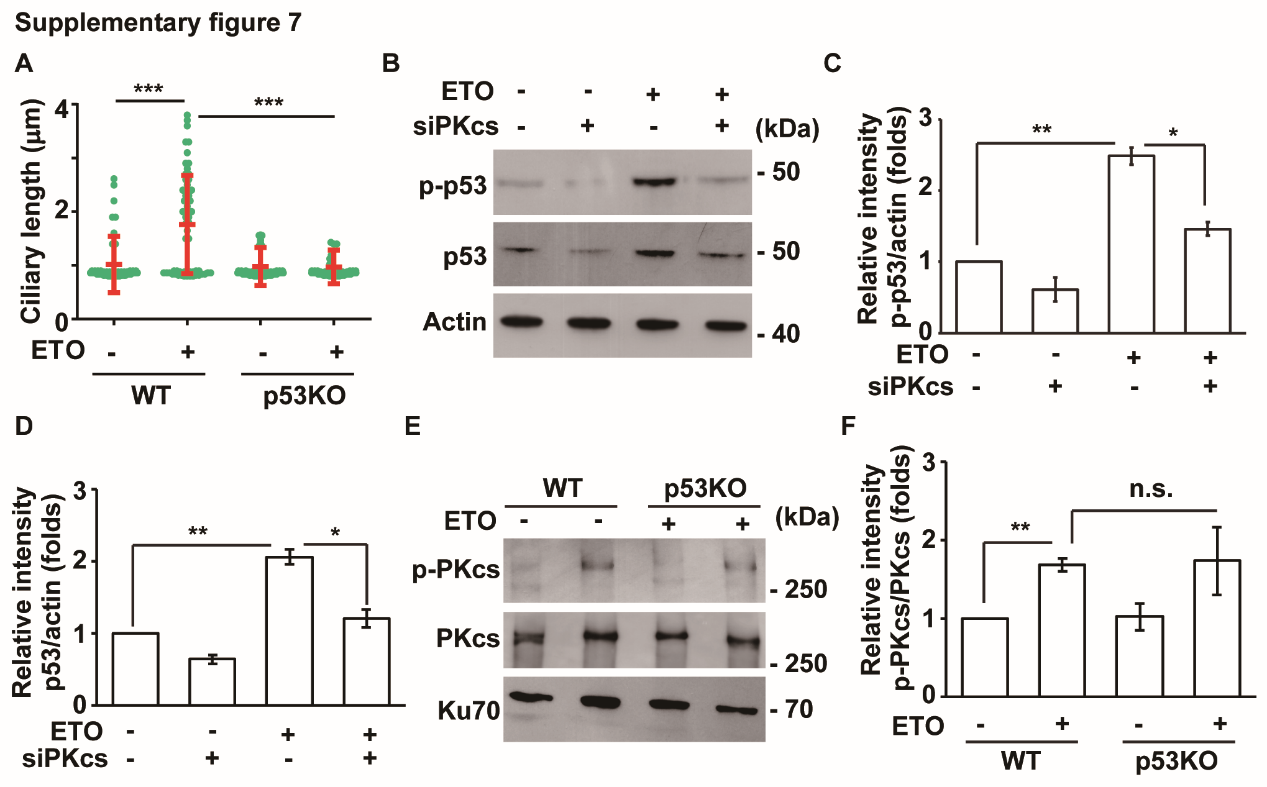


**Supplementary figure 7. DNA-PK activates p53 upon ETO treatment.**

(A) Quantitative results of the length of ciliated wild-type (WT) or p53 knockout (p53KO) RPE1 cells in the absence or presence of ETO. (B-D) Depletion of DNA-PKcs reduced ETO-activated p53. These results are mean +/- SD of three independent experiments; more than 100 cells were counted in each individual group. (B) Extracts of control or DNA-PKcs-deficient (siPKcs) RPE1 cells in the presence of ETO were analyzed by immunoblotting with antibodies against phosphorylated p53 (p-p53), p53, and actin. (C-D) Quantitative results of relative intensity of p-p53/actin (C) and p53/actin (D) of (B). All ETO-treated data were normalized to the data without ETO treatment. (E-F) Knockout of p53 did not reduce DNA-PK activation in ETO-treated RPE1 cells. (E) Extracts of wild-type or p53 knockout RPE1 cells in the presence of ETO were analyzed by immunoblotting with antibodies against phosphorylated DNA-PKcs (p-PKcs), PKcs, and Ku70. (F) Quantitative results of relative intensity of p-PKcs/PKcs of (F). * P<0.05; ** P<0.01; *** P<0.001; n.s. no significance.

**Supplementary figure 8.**


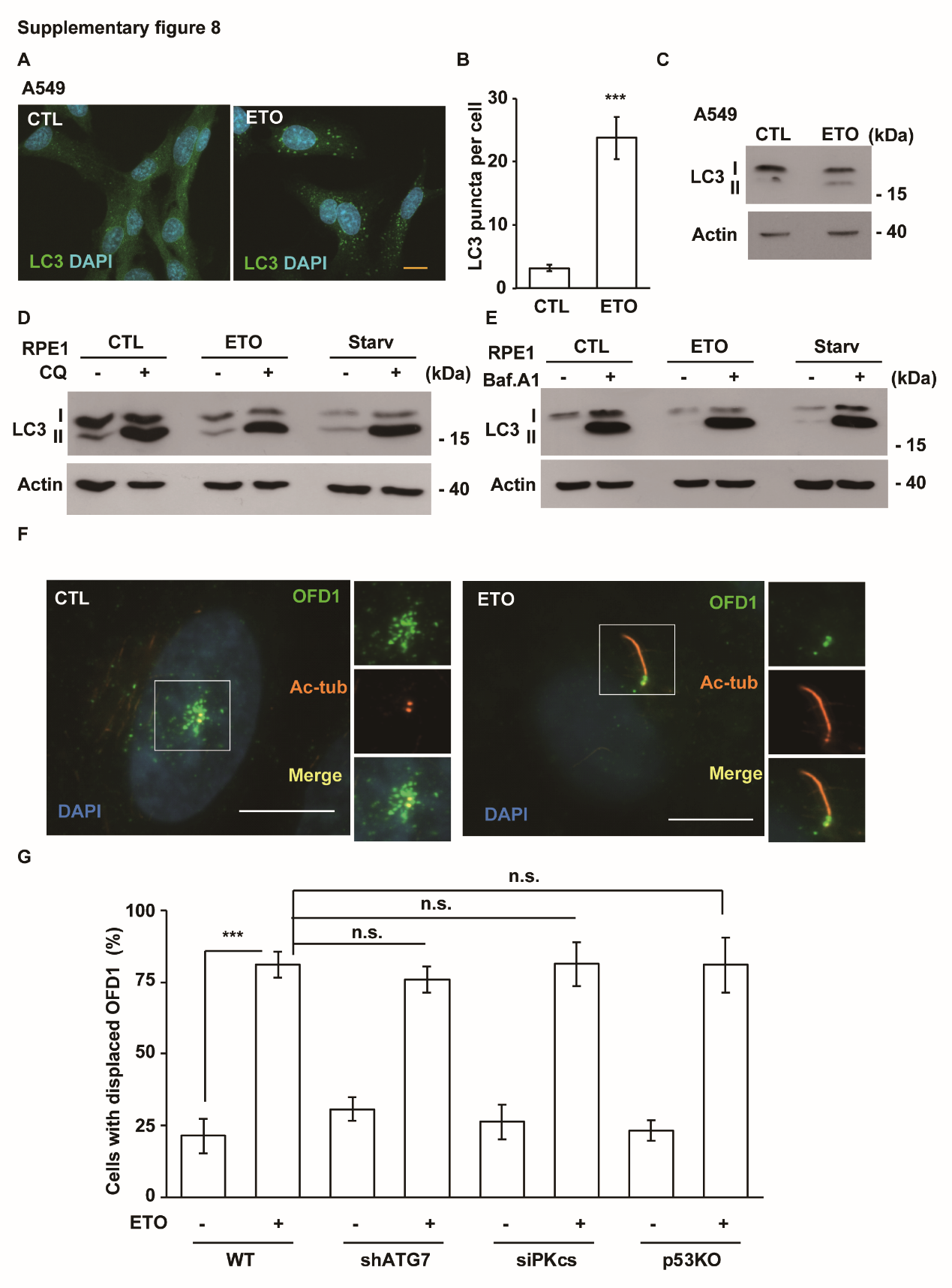


**Supplementary figure 8. ETO induces autophagy.**

(A-B) LC3 puncta were increased in ETO-treated A549 cells as shown by immunostaining with an antibody against LC3. DNA was stained with DAPI (blue). Scale bar, 10 µm. (B) Quantitative results of LC3 puncta per cell of A549 cells upon ETO treatment for 24 h. (C) Extracts of ETO-treated A549 cells were analyzed by immunoblotting with antibodies against LC3 and actin. (D-E) ETO treatment induced autophagic flux. Extracts of ETO treated RPE1 cells in the presence or absence of chloroquine (CQ; D) or bafilomycin-A1 (Baf. A1; E) or under serum starvation (Starv) for 24 h were analyzed with antibodies against LC3 and actin. (F) Displacement of OFD1 from centriolar satellites upon ETO treatment was analyzed by immunostaining with antibodies against acetylated tubulin (Ac-tub) and OFD1. DNA was stained with DAPI (blue). Scale bar, 5 µm. (G) DNA-PK-p53 cascade and autophagy did not regulate OFD1 displacement. Quantitative results of population of cells with displaced OFD1 of wild-type (WT), ATG7- (shATG7), DNA-PK- (siPKcs) or p53 knockout (p53KO) cells in the presence or absence of ETO. *** P<0.001; n.s. no significance.

**Supplementary figure 9.**


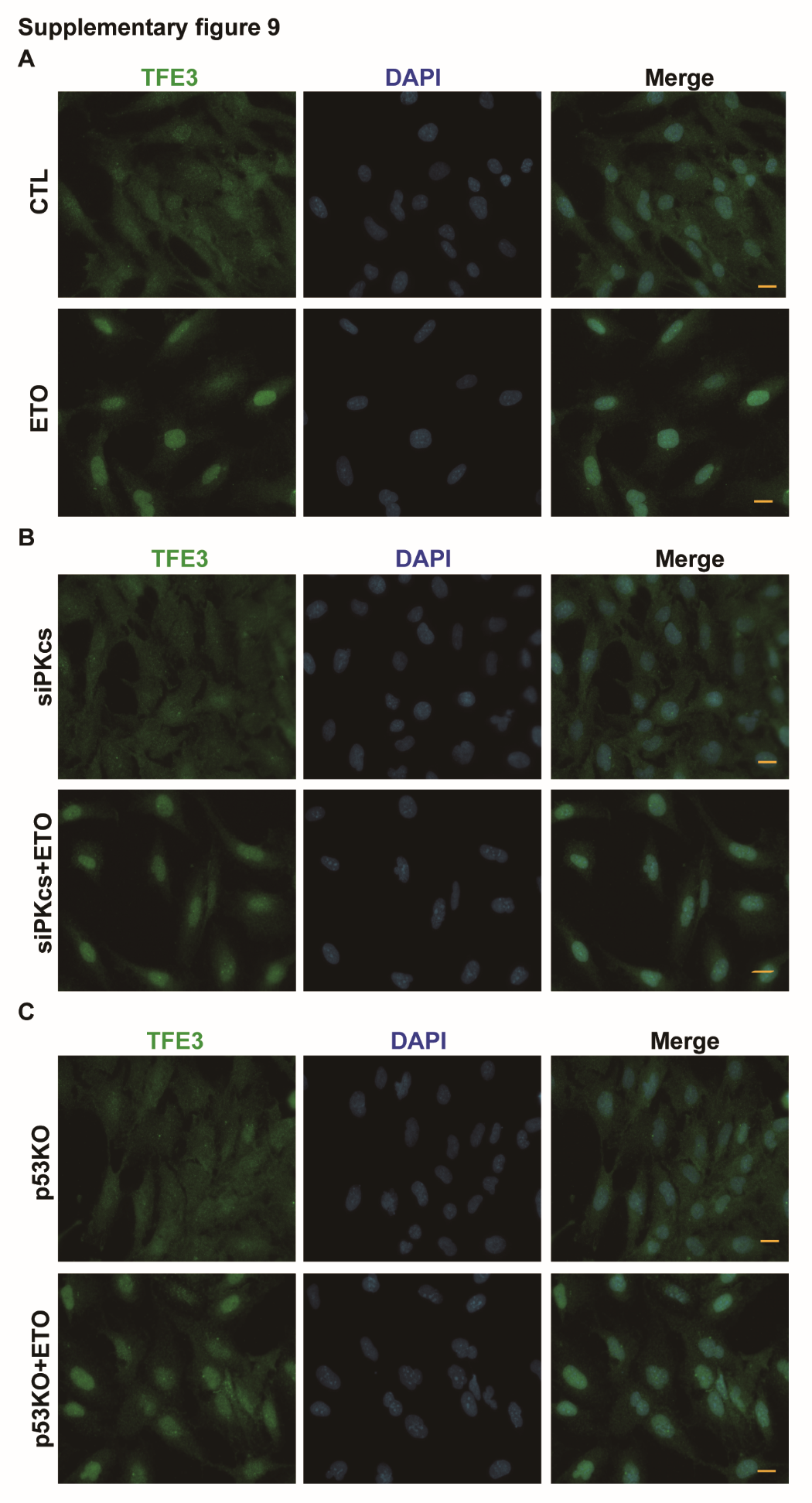


**Supplementary figure 9. DNA-PK and p53 do not regulate TFE3 activation.**

(A-C) Immunostaining of wild-type (A), DNA-PK- (siPKcs), or p53- (p53KO) deficient cells in the absence (upper panels) or presence (lower panels) of ETO with antibody against TFE3. DNA was stained with DAPI (blue). Scale bar, 10 µm.

**Supplementary figure 10.**


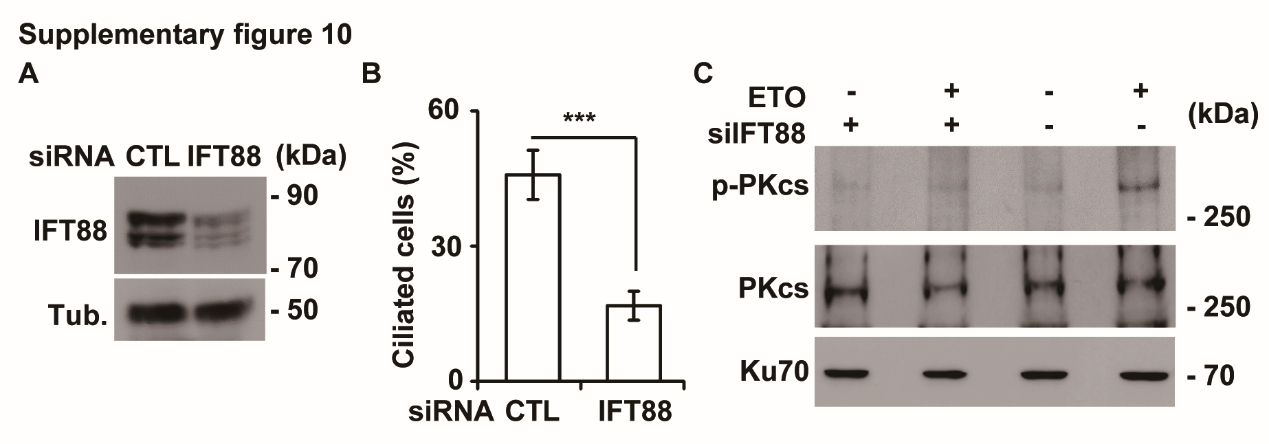


**Supplementary figure 10. The primary cilium maintains the ETO-induced DNA damage response.**

(A-B) Inhibition of ciliogenesis decreased ETO-induced DNA-PK activation. (A) Extracts of RPE1 cells transfected with siRNA against IFT88 were analyzed by immunoblotting with antibodies against IFT88 and tubulin (Tub.). (B) Quantitative results of the frequency of ciliated IFT88-deficient RPE1 cells in the presence of ETO. The results are presented as the mean +/- SD of three independent experiments; more than 100 cells were counted in each individual group. *** P<0.001. (C) Extracts of RPE1 cells transfected with siRNA against IFT88 in the presence or absence of ETO were analyzed by immunoblotting with antibodies against phosphorylated DNA-PKcs (p-PKcs), DNA-PKcs (PKcs), and Ku70.

**Supplementary figure 11.**


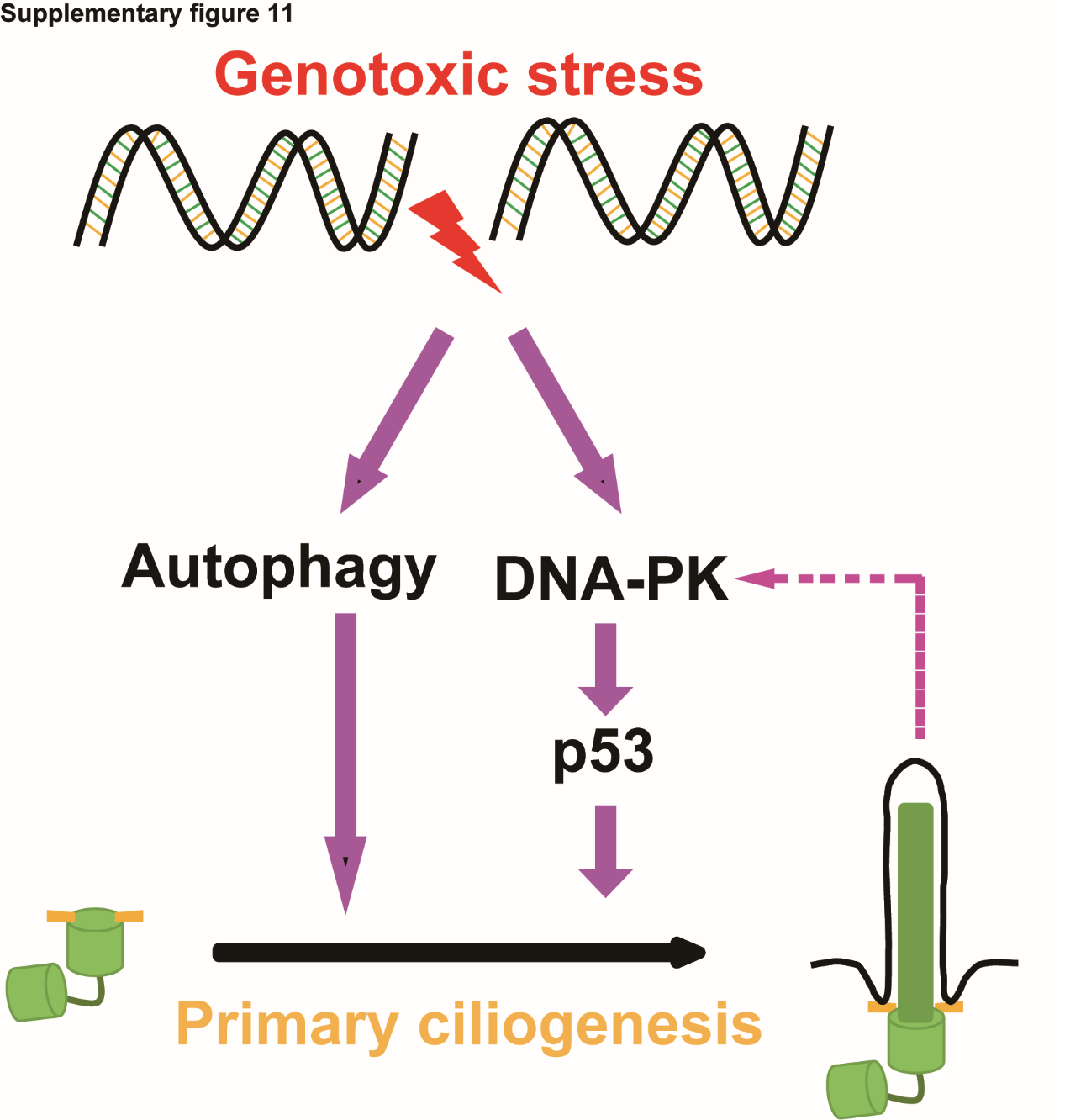


**Supplementary figure 11. A ﬂow chart depicting the molecular mechanism by which genotoxic stress induces primary ciliogenesis.**

Upon genotoxic stress, DNA-PK-p53 and autophagy was activated. Theses signaling cascade facilitated primary cilia formation. Interestingly, primary cilia further maintained DNA-PK activation. Thus, primary cilium maintains the ETO-induced DNA damage response.
