# Supplementary figures and images for: Genotoxic stress-activated DNA-PK-p53 cascade and autophagy cooperatively induce ciliogenesis to maintain the DNA damage response
Source: Cell Death Differ. 2021 Jan 18;28(6):1865–79. doi: 10.1038/s41418-020-00713-8 (PMC8184926; doi:10.1038/s41418-020-00713-8)

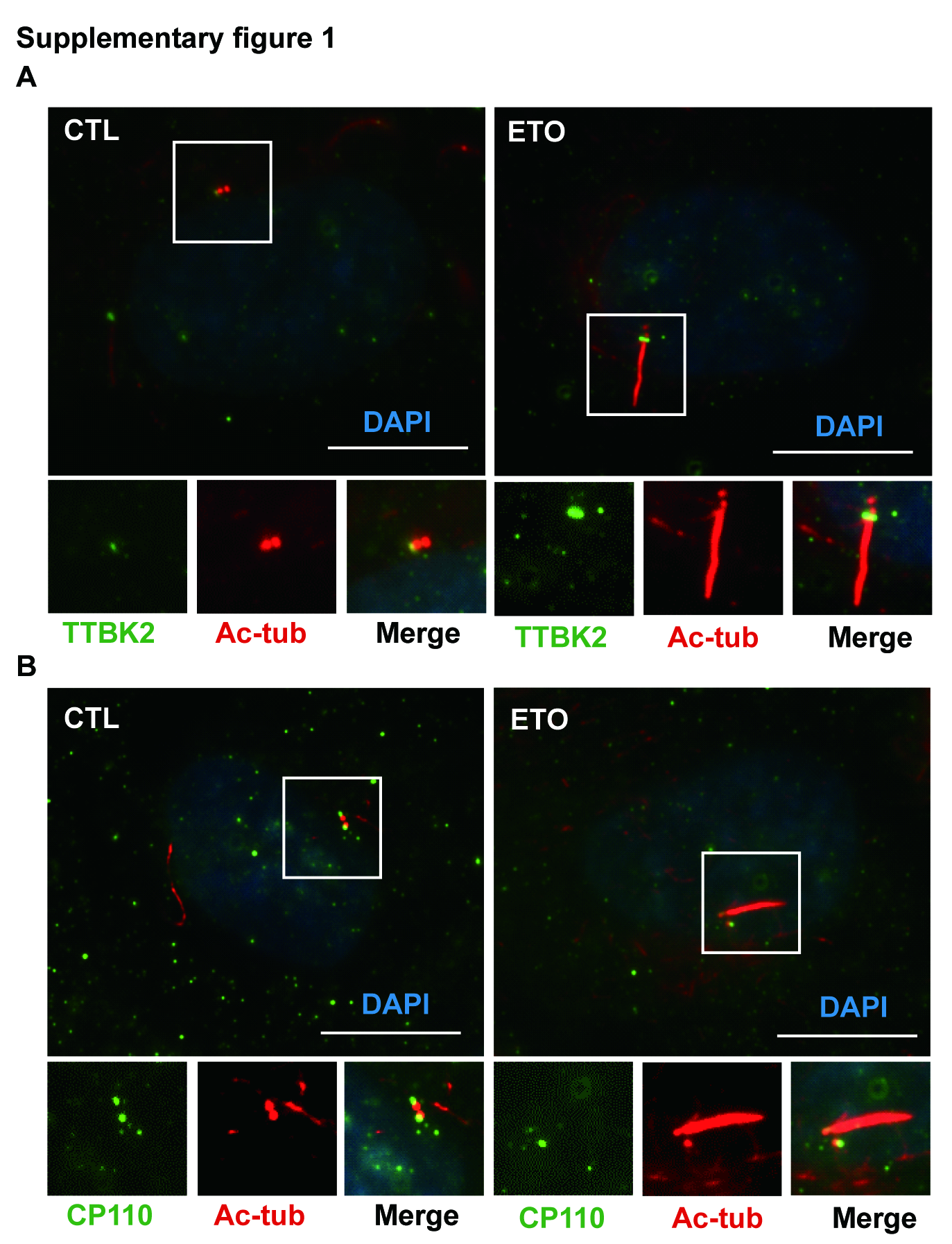

Supplement: Supplementary file 2 — Supplementary figure 1 [file 41418_2020_713_MOESM2_ESM.tif]

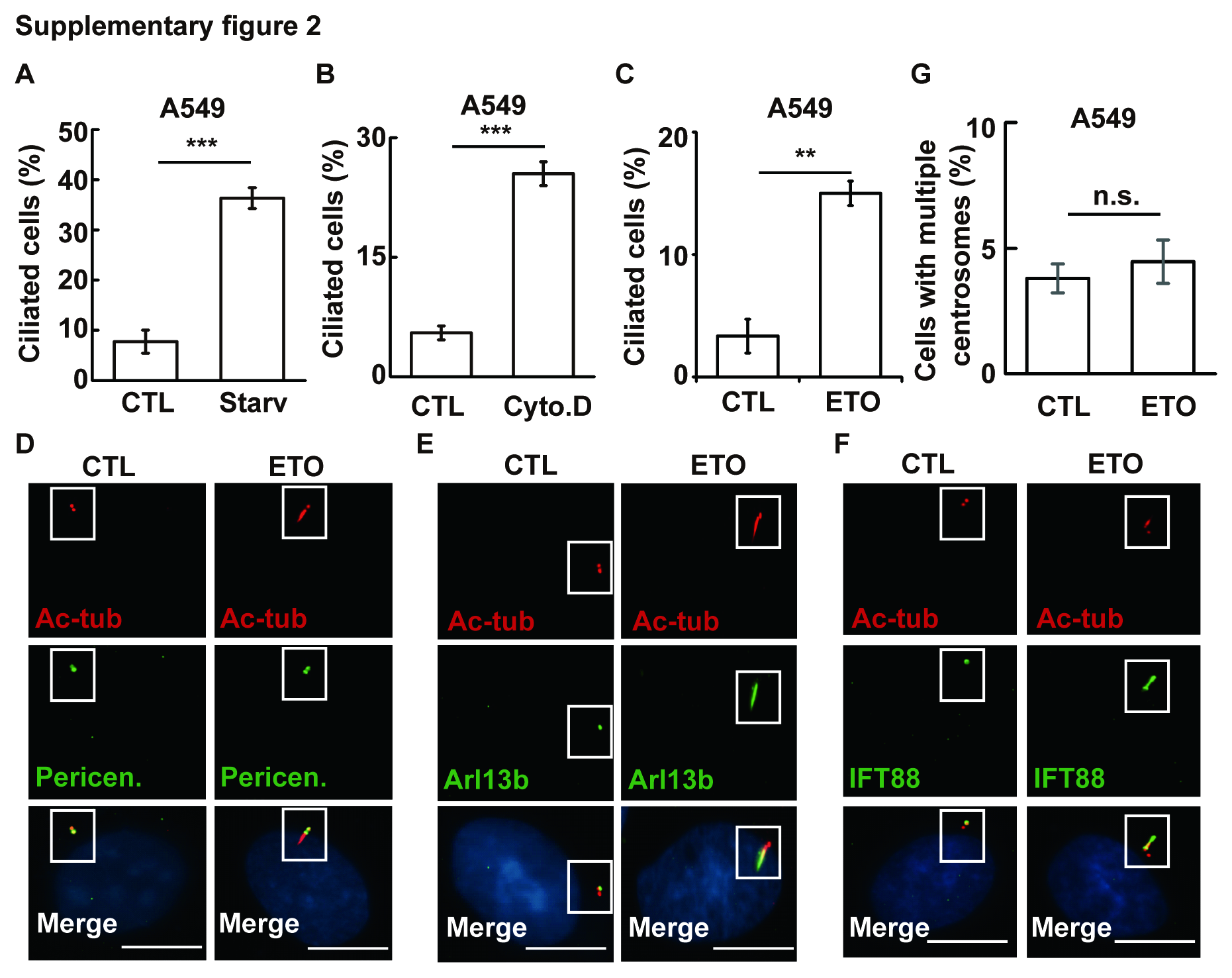

Supplement: Supplementary file 3 — Supplementary figure 2 [file 41418_2020_713_MOESM3_ESM.tif]

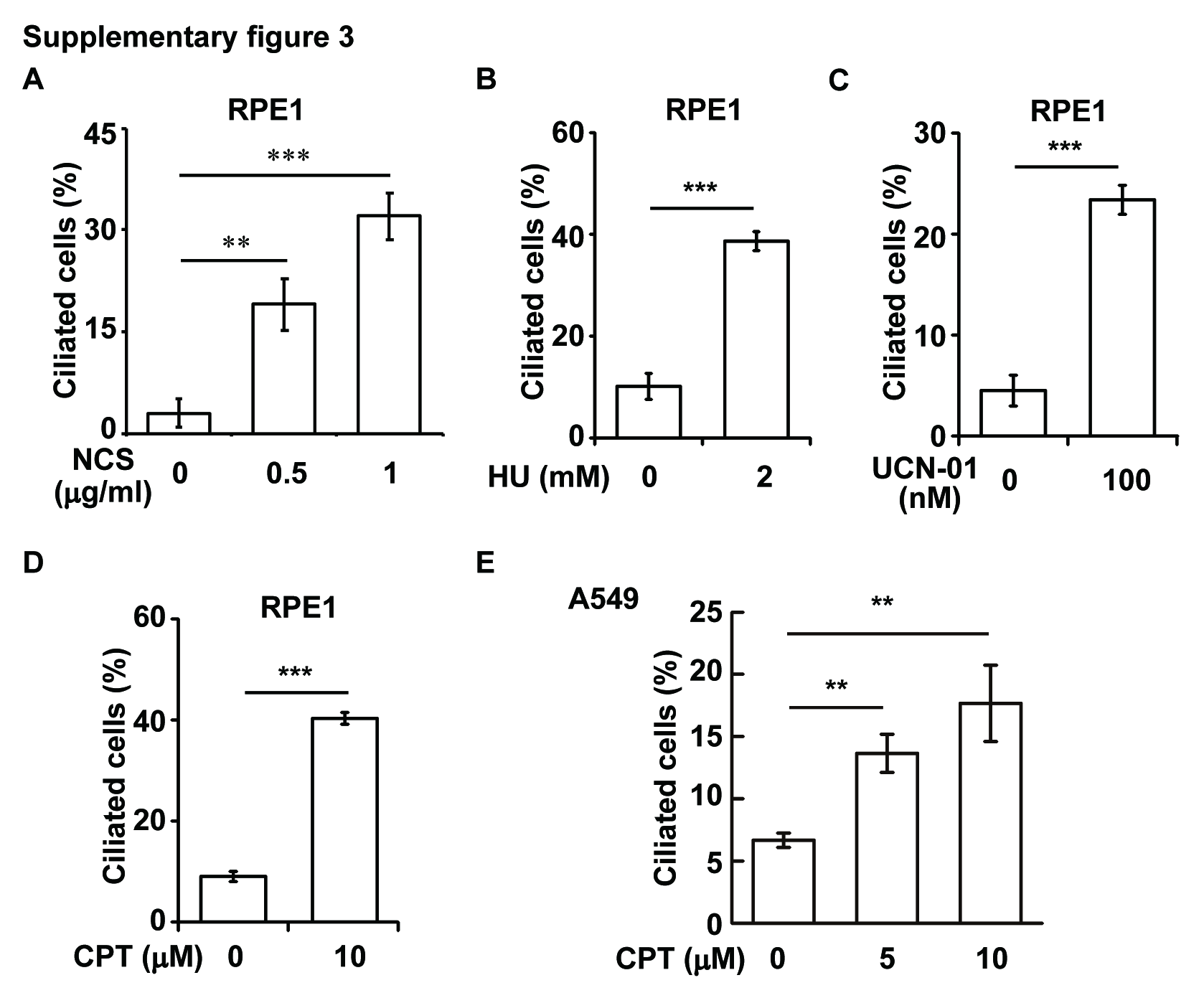

Supplement: Supplementary file 4 — Supplementary figure 3 [file 41418_2020_713_MOESM4_ESM.tif]

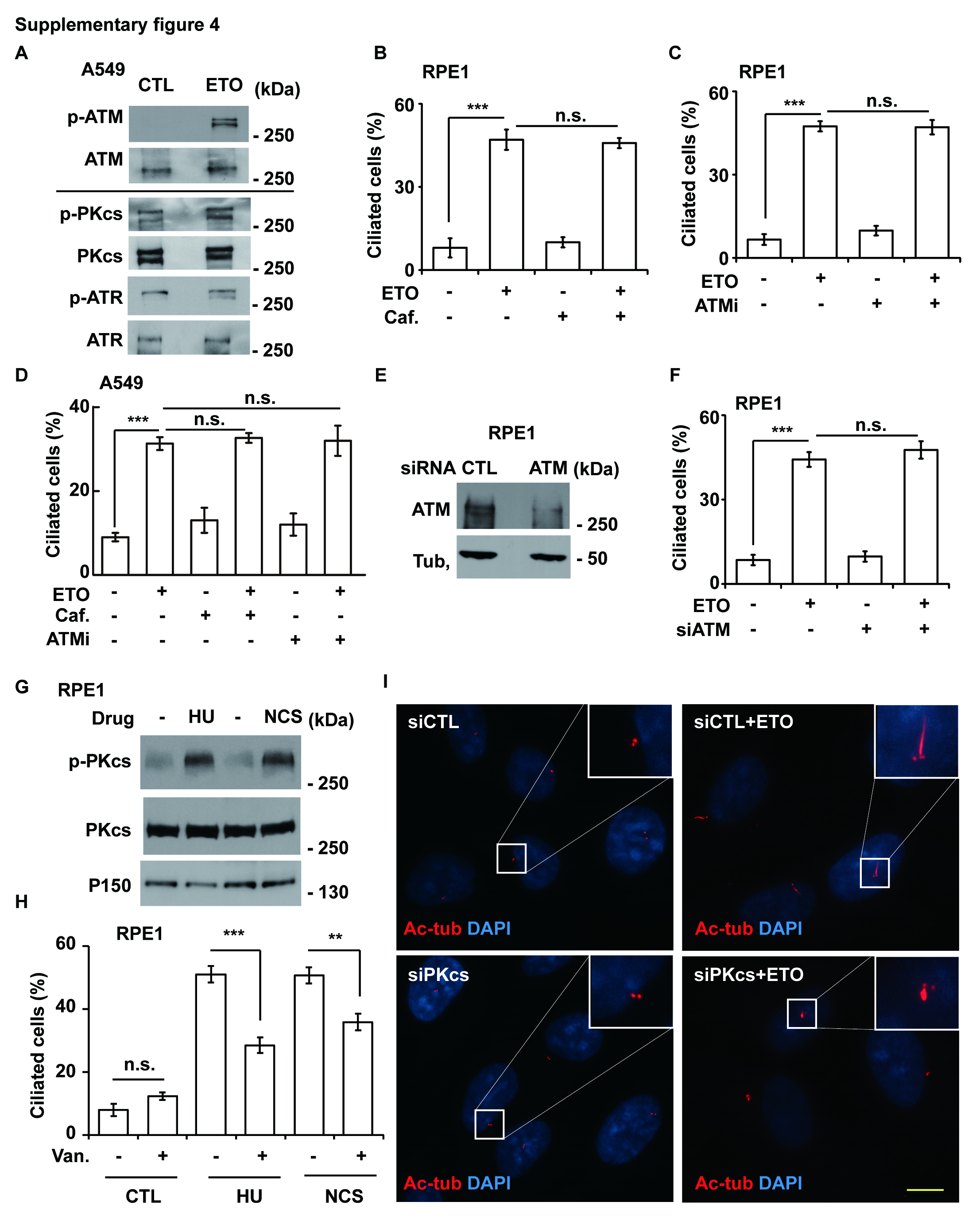

Supplement: Supplementary file 5 — Supplementary figure 4 [file 41418_2020_713_MOESM5_ESM.tif]

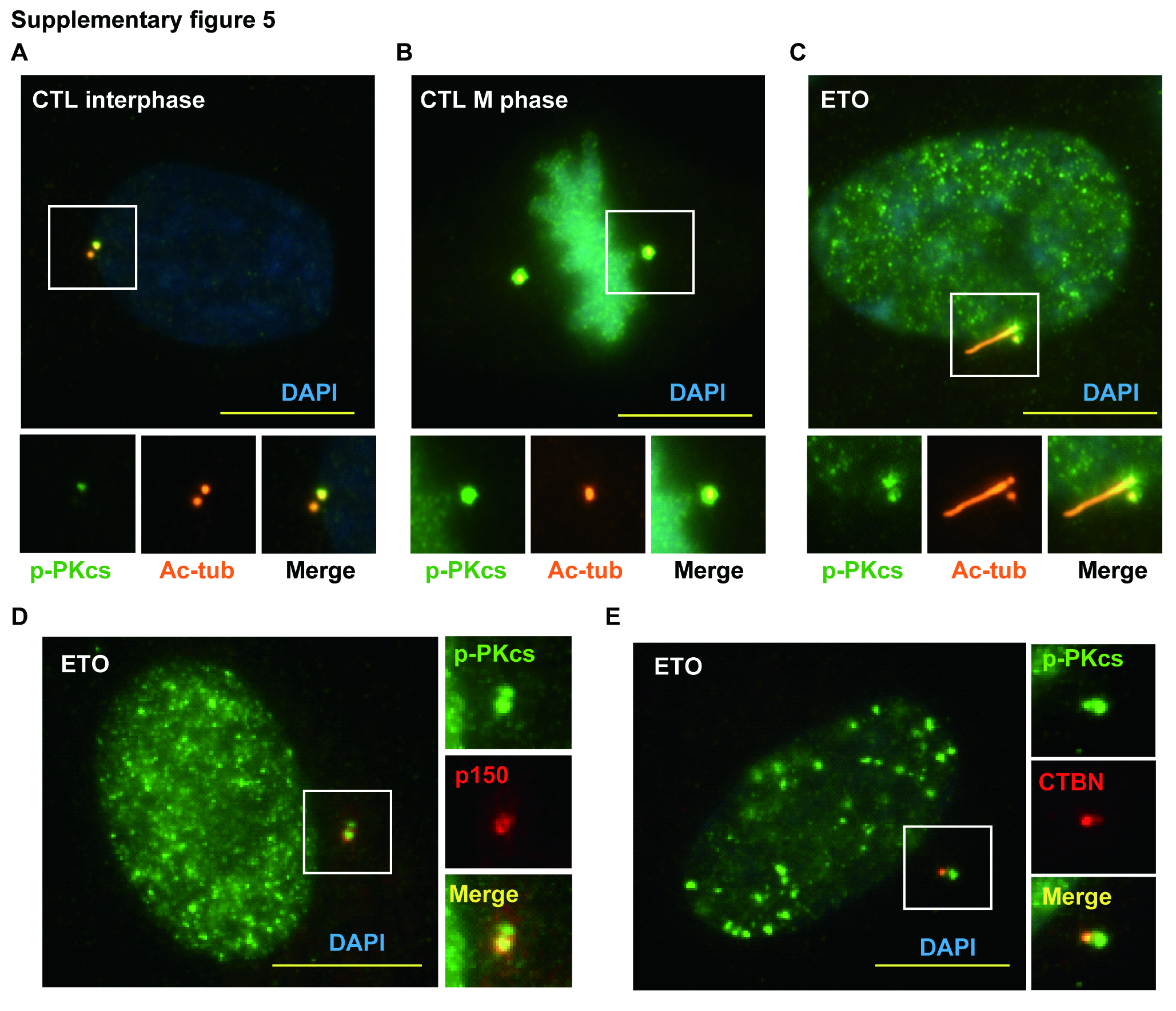

Supplement: Supplementary file 6 — Supplementary figure 5 [file 41418_2020_713_MOESM6_ESM.tif]

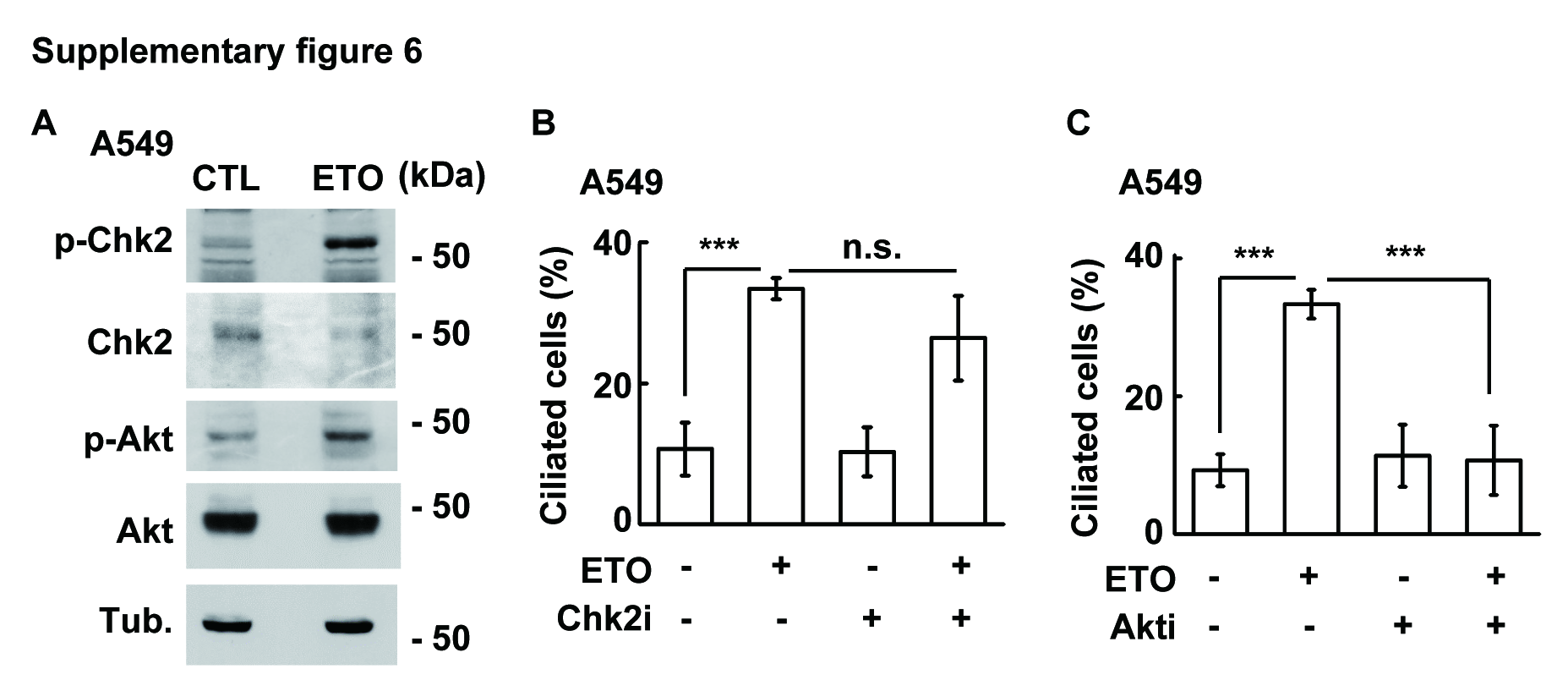

Supplement: Supplementary file 7 — Supplementary figure 6 [file 41418_2020_713_MOESM7_ESM.tif]

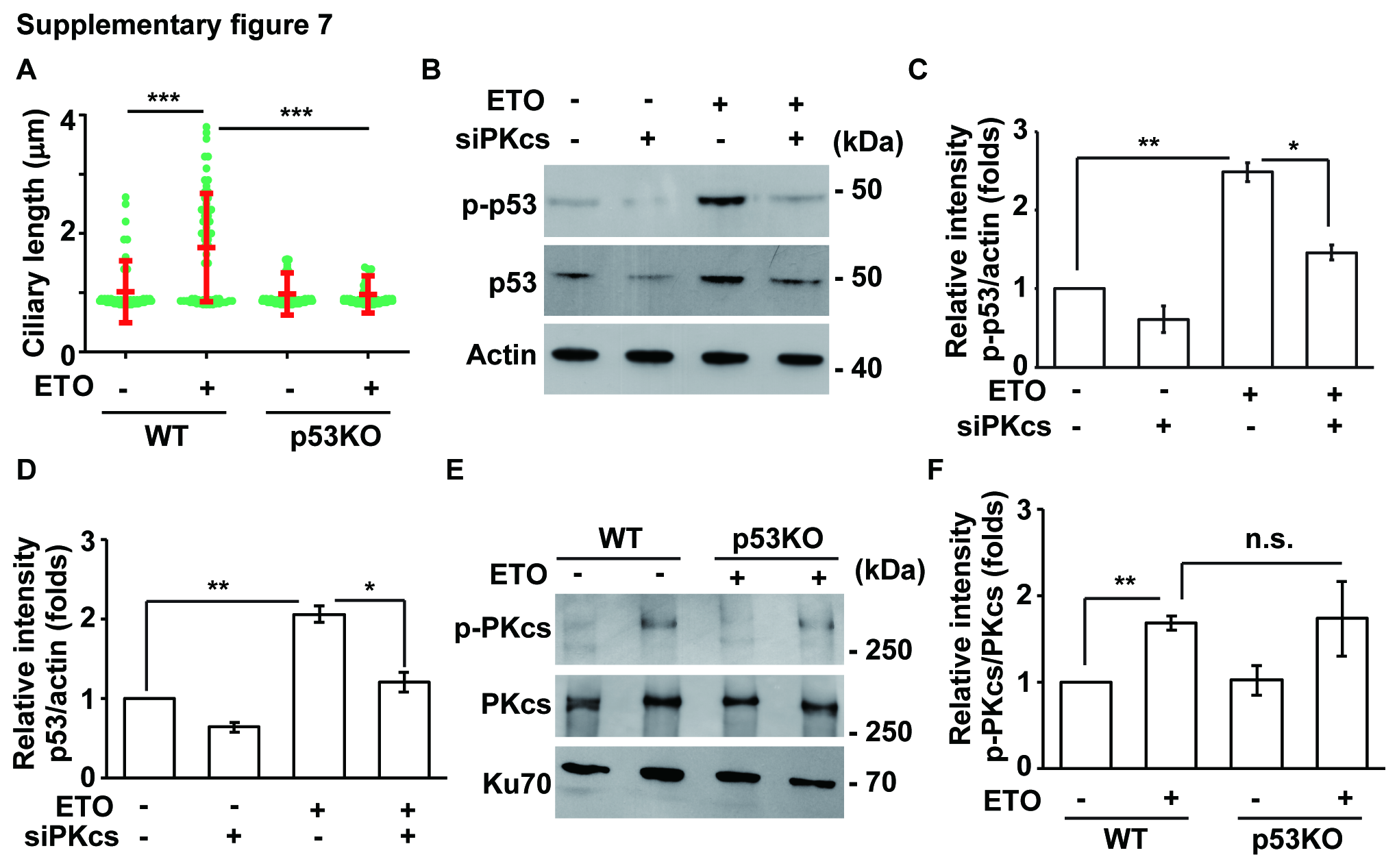

Supplement: Supplementary file 8 — Supplementary figure 7 [file 41418_2020_713_MOESM8_ESM.tif]

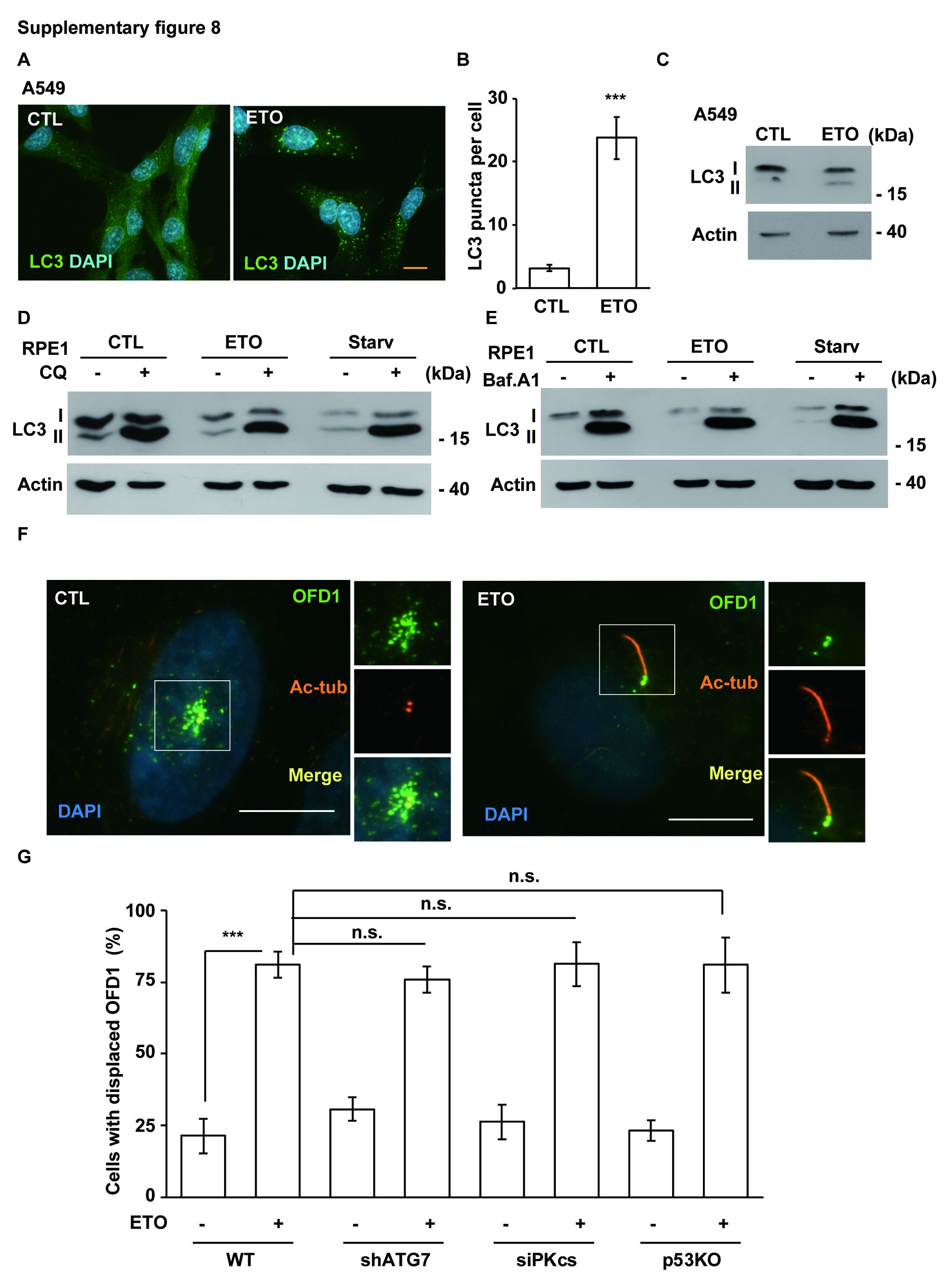

Supplement: Supplementary file 9 — Supplementary figure 8 [file 41418_2020_713_MOESM9_ESM.tif]

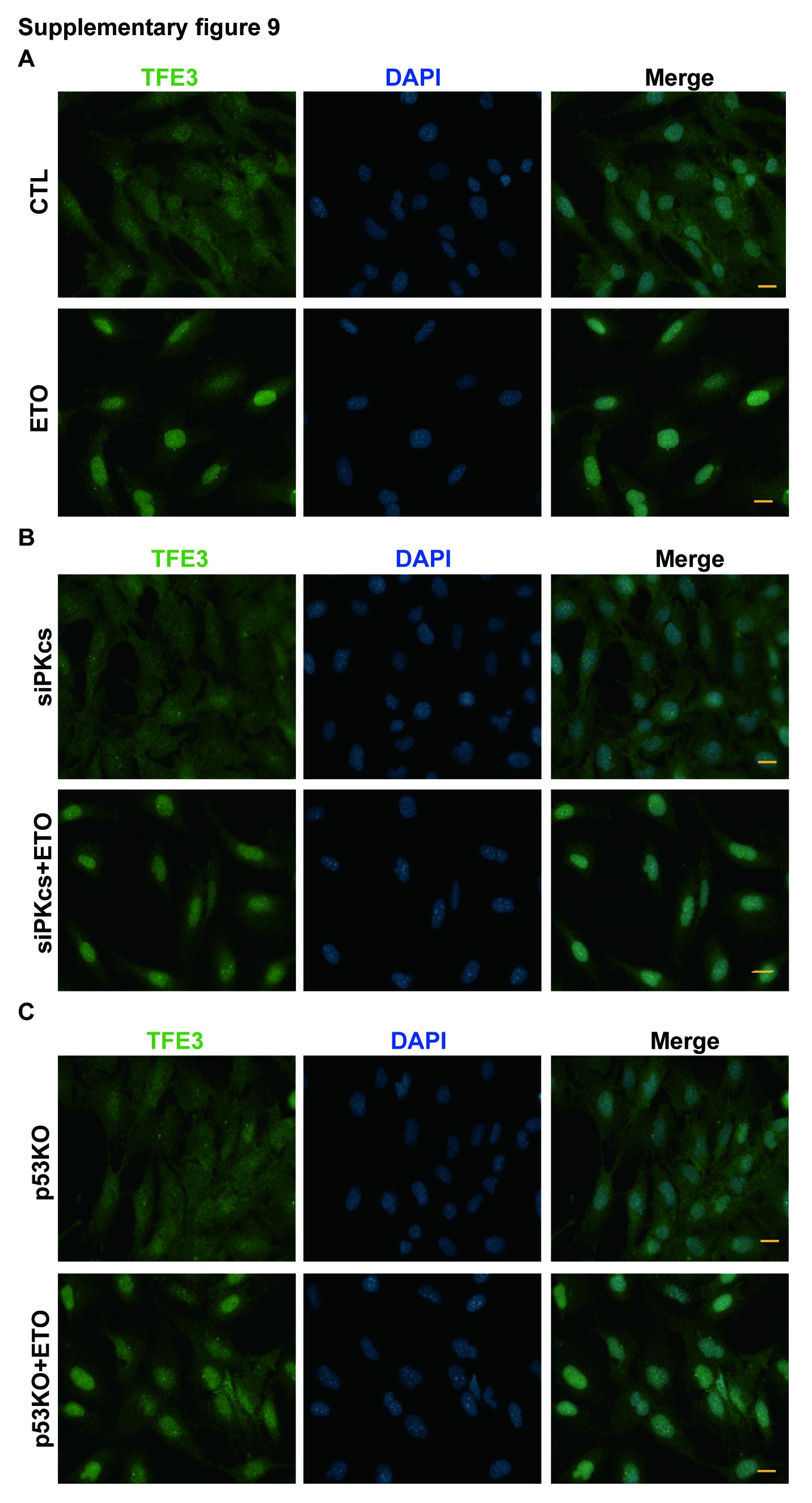

Supplement: Supplementary file 10 — Supplementary figure 9 [file 41418_2020_713_MOESM10_ESM.tif]

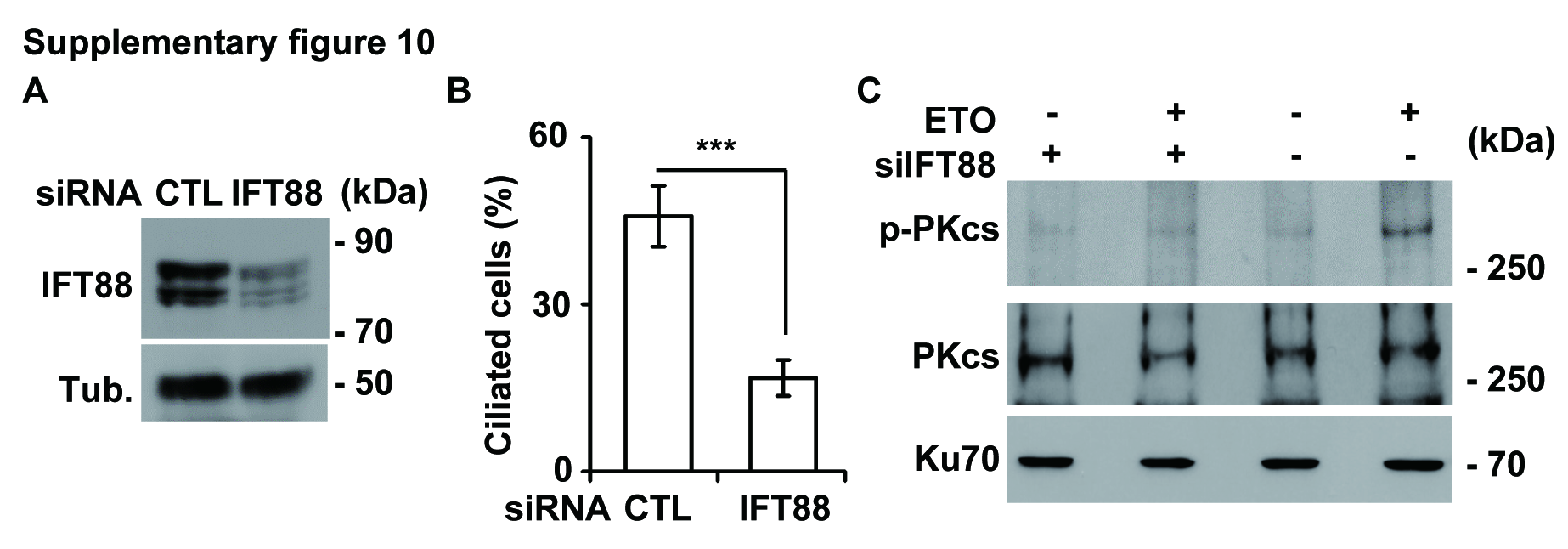

Supplement: Supplementary file 11 — Supplementary figure 10 [file 41418_2020_713_MOESM11_ESM.tif]

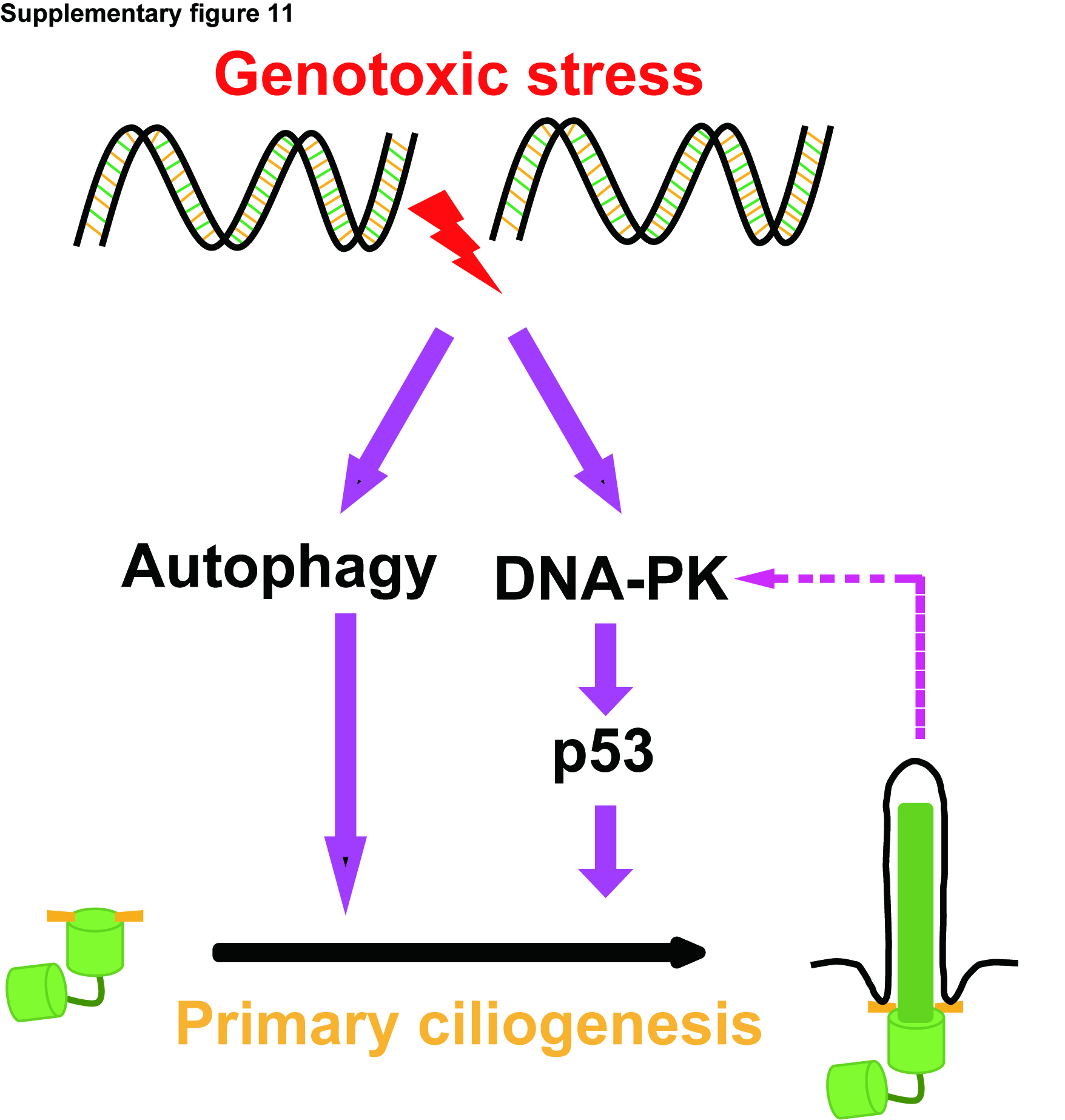

Supplement: Supplementary file 12 — Supplementary figure 11 [file 41418_2020_713_MOESM12_ESM.tif]
